# LncRNA NR_003508 Boosts *Pseudomonas aeruginosa*-Induced Autophagy by Facilitating the Conversion of LC3-I to LC3-II and p62 Degradation

**DOI:** 10.3390/cells15141306

**Published:** 2026-07-21

**Authors:** Li Liu, Jiaxue Zhang, Guangcun Deng, Xiaoling Wu

**Affiliations:** 1 School of Life Science, Ningxia University, Yinchuan 750021, China; l13895378870@163.com (L.L.); zhangjx8786@163.com (J.Z.); 2 Key Laboratory of Ministry of Education for Protection and Utilization of Special Biological Resources in Western China, Ningxia University, Yinchuan 750021, China

**Keywords:** LncRNA NR_003508, *Pseudomonas aeruginosa* (PA), autophagosome, LC3, p62

## Abstract

**Highlights:**

**What are the main findings?**

- LncRNA NR_003508 activates autophagy by promoting autophagosomes formation, thereby partly clearing *Pseudomonas aeruginosa*.
- LncRNA NR_003508 facilitates the conversion of LC3I to LC3II, a critical step in autophagosome formation, by targeting miR-344i. Simultaneously, it accelerates p62 degradation by sponging miR-344g-5p.

**What are the implications of the main findings?**

- As a member of long non-coding RNAs, LncRNA NR_003508 can promote cell autophagy and eliminate pathogenic bacteria.
- LncRNA NR_003508 is involved in boosting autophagy, indicating it may serve as a a theoretical basis and experimental support for understanding the mechanisms underlying *Pseudomonas aeruginosa* infection.

**Abstract:**

LncRNA NR_003508 has been implicated in lipopolysaccharide (LPS)-induced acute respiratory distress syndrome. Yet, the involvement of LncRNA NR_003508 in *Pseudomonas aeruginosa* (PA)-induced autophagy remains elusive. This study aims to reveal the underlying mechanisms of LncRNA NR_003508 in PA-induced autophagy, thereby enhancing the basic research content of the pathogenesis of PA infection. Fluorescence in situ hybridization (FISH) probes and q-PCR revealed that PA infection induced macrophage autophagy accompanied by increasing LncRNA NR_003508, suggesting that LncRNA NR_003508 may participate in competing endogenous RNA (ceRNA)-mediated regulatory networks. Concurrently, autophagic clearance of PA requires LncRNA NR_003508. Then, we used the Gene Expression Omnibus (GEO) database to find that LC3 was considered a core protein in PA-infected autophagy. Furthermore, LncRNA NR_003508 promoted the formation of autophagosomes, which played a positive role in PA clearance. Functionally, bioinformatics analysis and luciferase reporter assays demonstrated that LncRNA NR_003508 promotes the conversion of LC3I to LC3II by sponging miR-344i and facilitates p62 degradation by binding to miR-344g-5p. Taken together, our data established that LncRNA NR_003508 enhanced the formation of autophagosomes induced by PA infection via the miR-344i/LC3 and miR-344g-5p/p62 axes, partly boosting the clearance of PA and providing a theoretical basis and experimental support for understanding the mechanisms underlying PA infection.

## 1. Introduction

*Pseudomonas aeruginosa* (PA) frequently acts as an opportunistic pathogen in clinical environments. Upon infection, it can cause various diseases, including cystic fibrosis, pneumonia, and sepsis [1]. Given its high prevalence, associated mortality, and growing resistance to multiple antibiotics, PA infection has emerged as a major challenge in both clinical treatment and infection control [2]. Therefore, elucidating the underlying mechanisms of PA infection is of critical importance. Upon invasion, macrophages, as both primary target cells and key effectors of immune defense, initiate a series of immune responses, such as autophagy, apoptosis, and necrosis, to eliminate the pathogen and maintain cellular homeostasis. However, through the process of co-evolution with host cells, PA has created multiple methods to avoid and weaken immune responses, such as biofilm formation and secreting virulence factors [3]. Despite these adaptations, the regulatory mechanisms linking PA infection and autophagy remain highly complex. Long non-coding RNAs (LncRNAs), which are naturally occurring RNAs longer than 200 nucleotides, have been confirmed to regulate autophagy [4].

Autophagy can be activated in response to various pathological and cellular stress conditions. LncRNAs and microRNAs (miRNAs) interactions have been widely observed in autophagy across multiple disease settings [5]. Notably, the concept of competing endogenous RNA (ceRNA) introduces a novel mechanism of gene regulation, wherein LncRNAs behave like molecular sponges that soak up miRNAs, competing with miRNAs for binding and influencing the expression of their target genes [6,7]. MiRNAs, which are small non-coding RNAs, control the translation efficiency or post-transcriptional breakdown of target mRNA by attaching to the 3′-untranslated region (3′-UTR), which is a key regulatory domain in gene expression [8,9]. Furthermore, accumulating evidence showed that LncRNAs and miRNAs work together to form a LncRNA-miRNA-mRNA network, which is indispensable for the initiation, progression, and termination of autophagy [10,11].

Within the framework of the LncRNA-miRNA-mRNA axis targeting autophagy-related proteins, the interaction between lncRNA SP100-AS1 and miR-622 has been discovered, which targets ATG3 mRNA and promotes autophagic activity [12]. In pathogen infections, LncRNAs contribute to host immune defense and pathogen clearance by regulating autophagy. For instance, LncRNA DANCR fostered the survival of H37Ra by absorbing miR-1301-3p/miR-5194 [13]. With regard to autophagy-related signaling pathways, ncRNA Cancer Susceptibility Candidate 2 (CASC2) exhibited aberrant expression, affecting colon cancer cell autophagy by capturing miR-19a through the NF-κB pathway [14]. Moreover, a recently identified lncRNA, called 220, regulated autophagy and apoptosis through the miR-5101/PI3K pathway in mice with liver injury induced by LPS endotoxemia [15]. We previously demonstrated that LncRNA NR_003508 contributes to the onset of acute respiratory distress syndrome caused by LPS [16]. Yet, its involvement in PA-induced autophagy remains to be elucidated. Hence, investigating the molecular pathways by which LncRNA NR_003508 controls autophagy might offer insights into its use as a therapeutic target for PA infection.

The research identified the role of LncRNA NR_003508 in autophagy triggered by PA. Mechanistically, our findings revealed that LncRNA NR_003508 stimulated the formation of autophagosomes and boosted the clearance of PA via the miR-344i/LC3 and miR-344g-5p/p62 axes. The results highlighted the significant regulatory role of LncRNA NR_003508 in PA infection.

## 2. Materials and Methods

### 2.1. Ethics and Animals

Female C57BL/6J mice (6–8 weeks old; 20–25 g) free of specific pathogens were obtained from Shanghai Biomodel Organism Science & Technology Development (Shanghai, China). And mice were first numbered and then randomly allocated to different groups according to the Python random module. And only the first author was aware of the group allocation at the different stages of the experiment. The mice were kept at a temperature of 22 ± 2 degrees Celsius with a relative humidity of 40–60%, following a 12 h cycle of light and darkness. Mice were acclimated to the new environment (Ningxia University) for one week before the experiment began. Throughout the experiment, the mice had unrestricted access to regular food and water. Following intratracheal administration of 1 × PBS or PA (1 × 10^6^ CFU/mouse), the mice were euthanized in a CO_2_ chamber at various treatment intervals. Besides, to minimize potential confounders, the order of treatments was random.

**Figure 1:**
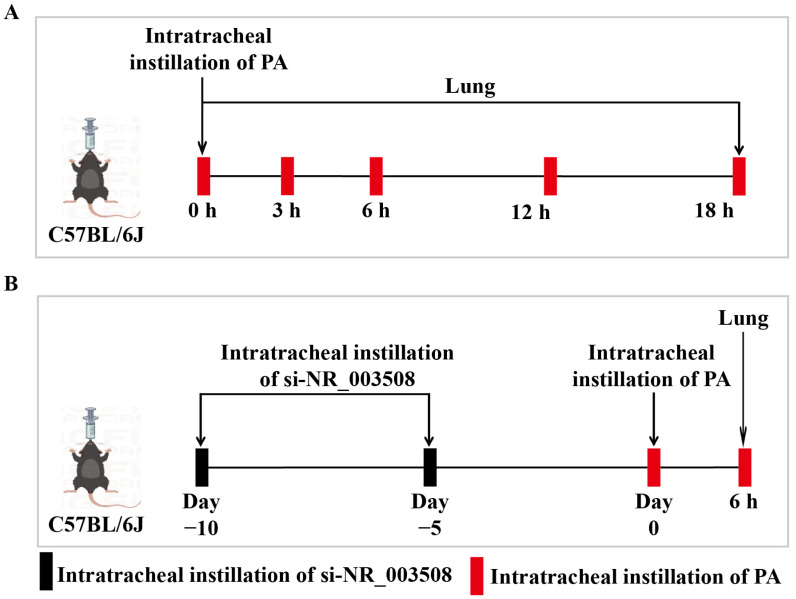
The specific treatments for C57BL/6J mice. (**A**) The first group with intratracheal instillation of PA. At different time points, peripheral blood as well as the mice’s lung tissues were collected simultaneously. (**B**) The second group with intratracheal instillation of si-NR_003508 and PA. Before PA infection, si-NR_003508 was instilled to knock down the target gene in the mice every 5 d for two times. Then peripheral blood and lung tissues were collected from mice 6 h after PA infection.

In total, the mouse experiments were divided into two groups of 5 mice each; Figure 1A shows the first group with 25 mice, and Figure 1B shows the second group with 20 mice, for a total of 45 mice in both groups. And then the first group set up 5 treatment groups, namely Ctr, 3, 6, 12, and 18, and the control group was Ctr, which was the intratracheal instillation of the 1 × PBS group; In the second group, 4 treatment groups were set up, namely NC, PA, si-NR_003508, and si-NR_003508 + PA, and the control group was NC (NC is a random sequence), which was the intratracheal instillation of the NC group. And 20 μL transfection reagent was added to each small interfering RNA Optical Density (OD) (1 OD refers to the amount corresponding to a solution with an absorbance of 1 at a wavelength of 260 nm and an optical path of 1 cm) to mix, and finally si-NR_003508 was prepared into a 50 pg/80 μL working solution with normal saline. Ten days before PA infection, NC, si-NR_003508 and si-NR_003508 + PA were instilled with NC and siRNA every five days to establish knockdown models, and then the mice were infected with 1 × 10^6^ CFU PA by intratracheal instillation for 6 h. Of these, they were excluded from the final analysis due to abnormal death, failure to meet baseline health criteria, and missing data records. The remaining three animals were included in the statistical analysis. According to the protocol approved by the ethics review board, the exclusion process was conducted. Animal experiments were conducted in accordance with the Chinese Council for the Care of Animals’ guidelines and approved by the Ningxia University Ethics Committee for Animal Research (NXU-ACAU-202409, 27 September 2024).

### 2.2. Transcriptomic Datasets Source

Data sets related to transcriptomics were acquired from the Gene Expression Omnibus (GEO) database. The dataset GSE295607 comprised pHBECs from 3 different CF donors (22F, 32G, 29H). In this article, we chose the untreated group, UT (from each donor, not exposed to PA or EV), and the PA group (pHBECs were exposed to the PA for 1 h).

### 2.3. Cell Culture and Bacterial Strain

RAW264.7 and 293T cells were grown in DMEM (Bobio, Zhejiang, China) supplemented with 10% FBS (Gibco, Waltham, MA, USA) in humid conditions (37 °C, 5% CO_2_). To isolate BMDMs, femurs and tibias of mice were flushed with 1 × PBS, then cultured in DMEM with 10% and 25 ng/mL macrophage M-CSF (MCE, California, USA). Then at 37 °C, 5% CO_2_ maintained for a week, and the culture medium is added once in the middle.

The *Pseudomonas aeruginosa* (PA) was cultivated with Luria-Bertani medium. The cells were subjected to transfection with a small interfering RNA for a duration of 24 h and were subsequently collected after being cultured at different time points at 20 Multiplicity of Infection (MOI) or different MOI for 4 h PA infection.

### 2.4. Insertion of Small Interfering RNA (siRNA) and Plasmid

GenePharma (Shanghai, China) designed and produced Si-NR_003508 along with negative controls (NC). The LncRNA NR_003508, LC3 and p62 dual luciferase plasmids were designed by us and constructed by Gene Optimal (Shanghai, China). (The sequences of the plasmid were compiled in Supplementary File S2) Cells were exposed to NC and three separate siRNAs whose sequences are described in our published paper [17]. Effective siRNA targeting sequences or plasmids were used for the transfection of RAW264.7 and 293T cells with Nulen PlusTrans^TM^ Transfection Reagent (Shanghai, China) at 37 °C. Further analysis of the transfected cells was conducted 24 h later.

### 2.5. Reverse Transcription-Polymerase Chain Reaction (q-PCR)

The total RNA from RAW264.7, BMDM, and C57BL/6J mouse lung tissues was extracted using TRIzol reagent (TIANGEN, Beijing, China). The synthesis of complementary strands to the total RNA of LncRNA NR_003508, Atg5, Atg7, Atg12, and p62 was performed using the PrimeScript RT kit from TaKaRa, Shiga, Japan. While miRNAs were used with the RevertAid RT Reverse Transcription Kit (Thermo, Waltham, MA, USA). The amount of total RNA or miRNA used for cDNA synthesis was 1 µg. For qPCR, 95 °C, 3 min (pre-denaturation step); 95 °C, 5 s and 60 °C, 30 s (40 cycles). Using 2^−ΔΔCt^ to calculate the relative levels of LncRNA NR_003508 and Autophagy factors adjusted by β-actin. The miRNAs were normalized with reference snRNA202. Table 1 displays the sequence of the primers.

### 2.6. Western Blot

In a 6-well plate, cells were placed at a concentration of 1 × 10^6^ cells per well, washed with 1 × PBS, and lysed for 20 min with protein extraction buffer from Keygen BioTech, Nanjing, China. Protein levels were assessed using a BCA test kit provided by Thermo Fisher Scientific, located in Waltham, MA, USA. Subsequently, the same 20–30 mg of proteins were then analyzed using SDS-PAGE and a PVDF membrane with an 8–10% resolving gel. To block the membranes, 5% nonfat milk was applied for 2 h, primary antibodies at 4 °C overnight, and secondary antibodies for 2 h. ECL (ABclonal, Wuhan, China) was used to visualize the protein bands, and Image J was employed for result analysis. The primary antibodies β-actin (Proteintech, Chicago, IL, USA; 66009-1-Ig; Dilution multiple 1:2000), Atg5 (Proteintech, Chicago, IL, USA; 10181-2-AP; Dilution multiple 1:1000), Atg7 (Proteintech, Chicago, IL, USA; 67341-1-Ig; Dilution multiple 1:1000), Atg12 (Abclonal, Wuhan, China; A22788; Dilution multiple 1:1000), LC3 (Abclonal, Wuhan, China; A12319; Dilution multiple 1:2500), and p62 (Proteintech, Chicago, IL, USA; 66184-1-Ig; Dilution multiple 1:2000) were utilized for the Western blot, along with HRP-conjugated Goat Anti-Rabbit/Mouse IgG(H + L) (Proteintech, Chicago, IL, USA; SA00001-2/-1; 1:5000 dilution).

### 2.7. Fluorescence in Situ Hybridization (FISH)

FISH was employed to verify where LncRNA NR_003508 is located within the cell. The fluorescence was C3-Fluorescein, and the sequence of the FISH fluorescent probes was shown in Table 2. Then the experiment was performed in accordance with the instructions of the in situ hybridization kit with fluorescence from GenePharma in Shanghai, China. The cells were visualized under a fluorescent microscope (Olympus, Tokyo, Japan).

### 2.8. RAW264.7 Cells Subcellular Fractionation

We first extracted total RNA from cells using RNAeasy^TM^ Plus Animal RNA lsolation Kit with Spin Column (Beyotime Biotechnology, Shanghai, China; R0032). The separation of cytoplasmic and nuclear fractions was achieved with the Nuclear/Cytosol Fractionation Kit (Phygene Biotechnology, Shanghai, China; PH1466). After isolation, the expression levels of glyceraldehyde-3-phosphate dehydrogenase (GAPDH), U6 and LncRNA NR_003508 were detected by qPCR. As a control, GAPDH was utilized for the cytoplasm and U6 for the nucleus.

### 2.9. Immunofluorescence Assay

3 × 10^5^ cells per well in a 12-well plate and transfected with LncRNA NR_003508 stylet, miRNA mimics, and LC3 and p62 Plasmids, then treated with or without PA for 4 h. Next, RAW264.7 or 293T cells underwent fixation for 30 min at room temperature with 4% paraformaldehyde, followed by a 30-min permeabilization using PBS with 0.5% Triton × −100. A 2 h incubation at 37 °C with primary antibodies (Dilution multiple 1:200) and secondary antibodies (Abcam, Cambridge, UK; ab150077; Dilution multiple 1:500). For 10 min, the nucleus was stained using DAPI from ZSGB-BIO in Beijing, China. A laser confocal microscope (Olympus, Tokyo, Japan) was used to capture the images.

### 2.10. Confocal Microscope Assay for Autophagy Flux

HanHeng Technology in Shanghai, China, supplied the autophagy indicator known as mRFP-GFP-LC3 adenoviral. In brief, RAW264.7 cells were transfected with si-NR_003508, Rapamycin (RAPA), and 3-Methyladenine (3-MA) for 24 h. Afterward, RAW264.7 cells were subjected to transduction using mRFP-GFP-LC3 adenoviral. Following 8 h of transfection, PA infection was carried out on the cells for 4 h. Autophagosomes (yellow dots) and autolysosomes (red dots) were finally visualized under a confocal microscope to evaluate the autophagy flux.

### 2.11. Cyto-ID Autophagy Detection

The cationic amphiphilic tracer dye Cyto-ID is used to specifically detect autophago(lyso)somes, and its quantification is possible through flow cytometry. Autophagy activation was quantified using flow cytometry, with Cyto-ID^®^ dye from Enzo Life Sciences (New York, NY, USA) binding to autophagosomes. Briefly, a total of 3 × 10^5^ RAW264.7 cells were placed in each well of 12-well plates. Following treatment with PA and si-NR_003508, the samples underwent washing with 1 × PBS. Then, cells were harvested with trypsin and prepared according to the manufacturer’s instructions. Each erythroid subpopulation’s autophagosome content was evaluated by measuring the geometrical mean of Cyto-ID (FITC). In the flow cytometry experiment, 3 × 10^4^ valid single-cell events were collected. The FL2 fluorescence channel was displayed on the X-axis, and on the Y-axis was Count, which is a linear coordinate, indicating the number of cells within the corresponding FL2 fluorescence intensity range. The gating was performed using a one-dimensional histogram rectangle gate, named M1, to separate the FL2 positive cell population.

### 2.12. Luciferase Reporter Assay

The luciferase reporter system was utilized to conduct luciferase reporter assays. Briefly, in HEK293T cells, transfection was performed using the wild type (WT) and mutant (Mut) of LncRNA NR_003508, together with the LC3/p62 sequence that has the miR-344i/miR-344g-5p binding site and luciferase reporter vectors (Gene Optimal, Shanghai, China) using Lipofectamine TM 3000 (Invitrogen, San Diego, CA, USA). miR-344i/miR-344g-5p mimics were manufactured by GenePharma (Shanghai, China). After 36 h, luciferase activity was assessed by the Dual Luciferase Reporter Assay System from Promega, Madison, WI, USA.

### 2.13. RNA Immunoprecipitation (RIP) Assay

The genes’ interaction was identified through a RIP assay utilizing a BeyoRIP^TM^ RIP Assay Kit (Protein A/G Agarose) (Beyotime, Shanghai, China). Ago2 (67934-1-Ig) antibody and IgG (AC005) were purchased from proteintech and ABclonal. After being collected by centrifugation, 293T cells (2 × 10^6^) were lysed in RIP lysis buffer. Then, the lysates from the cells were then divided into two equal sections and treated with 5 μg of antibody, rotating at 4 °C for the duration of the night. Using 20 μL DEPC water for elution, the enriched RNA can then be obtained for subsequent detection. Afterward, we employed q-PCR to individually measure the expression of LncRNA NR_003508, miR-344i, miR-346-3p, LC3, and p62 in the enriched RNA.

### 2.14. Hematoxylin and Eosin (HE) Staining

The lung tissues were fixed in 4% paraformaldehyde for a duration of 48 h and subsequently embedded in paraffin. Sections of paraffin, 7 μm thick, underwent deparaffinization and rehydration. Standard hematoxylin and eosin (HE) staining was applied to lung sections, which were then examined using an optical microscope in Tokyo, Japan. Histopathological lung injury was also evaluated.

### 2.15. Immunohistochemistry

In short, paraffin sections underwent deparaffinization, rehydration, and antigen repair. For IHC staining, the sections of the lung were then exposed to 3% hydrogen peroxide for 20 min. First, a 40 min blocking with 5% BSA was applied to the paraffin sections, then exposed to primary antibodies above at 4 °C overnight, and subsequently stained with secondary antibodies (Proteintech, Chicago, IL, USA; SA00004-1, SA00004-2; Dilution multiple 1:500) for 1 h at 37 °C. At room temperature, diaminobenzidine (DAB, G1212-200T, Servicebio, Wuhan, China) was applied for 1–3 min for coloration. Hematoxylin was used to counterstain the slides. Primary antibodies included Atg5 (Proteintech, Chicago, IL, USA; 10181-2-AP; Dilution multiple 1:1000), Atg7 (Proteintech, Chicago, IL, USA; 67341-1-Ig; Dilution multiple 1:1000), Atg12 (Abclonal, Wuhan, China; A22788; Dilution multiple 1:200), LC3 (Proteintech, Chicago, IL, USA; 14600-1-AP; Dilution multiple 1:200), and p62 (Proteintech, Chicago, IL, USA; 66184-1-Ig; Dilution multiple 1:2000).

### 2.16. Bacterial Load Determination

Mycobacterium tuberculosis load was evaluated by counting MOI. Samples were homogenized lightly with 0.5% Triton × −100 to disrupt aggregates, and serial dilutions of 10^−2^ to 10^−5^ were prepared. Then, 100 microliters of each sample (macrophage lysate and liver homogenate) were spread onto LB plates. Overnight incubation of plates at 37 °C was followed by counting the colony-forming units.

### 2.17. Statistical Analysis

GraphPad Prism 9.5 software (GraphPad, Santiago, WA, USA) was used to conduct the statistical analysis. The data were derived from a minimum of three separate experiments and are presented as mean ± SD. A *T*-test was used to compare two groups, while data from experiments involving three or more groups were analyzed using one-way ANOVA. A *p*-value of less than 0.05 was considered statistically significant for all tests.

## 3. Results

### 3.1. PA Infection Increases LncRNA NR_003508 Expression In Vivo and In Vitro

**Figure 2:**
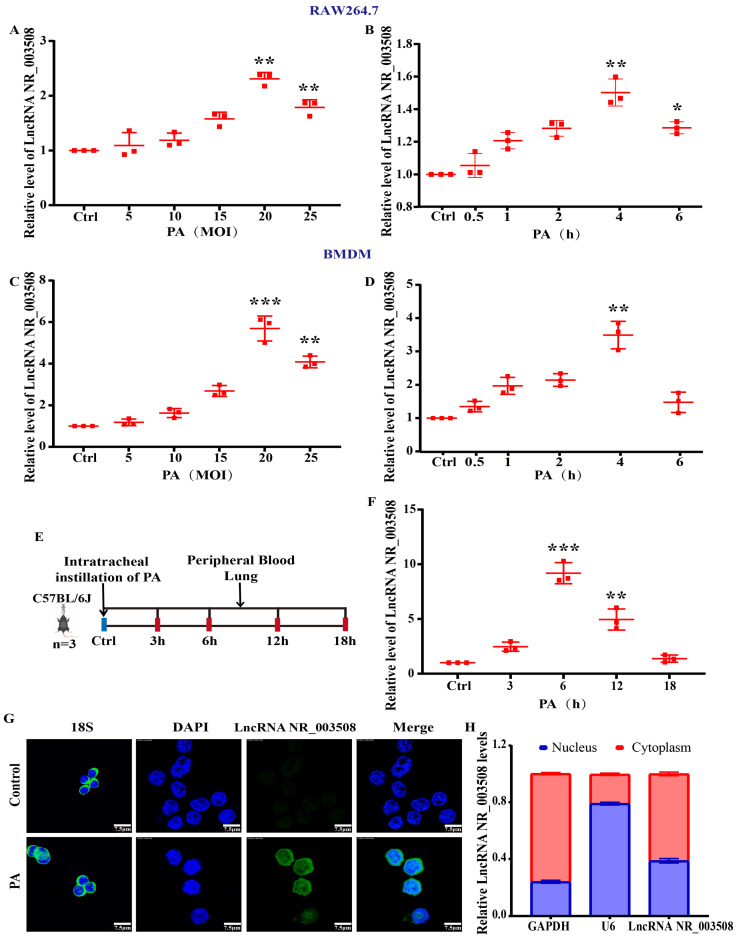
PA infection increases LncRNA NR_003508 expression in vivo and in vitro. (**A**,**B**) The expression levels of LncRNA NR_003508 in RAW264.7 cells were measured using q-PCR after exposure to PA at different MOI and varied time points in RAW264.7 (*n* = 3). * *p* < 0.05, ** *p* < 0.01 vs. the control cells. (**C**,**D**) In BMDM, the expression of cellular LncRNA NR_003508 was monitored via q-PCR following PA treatment at different MOI, and various doses and time points by q-PCR assay in BMDM (*n* = 3). ** *p* < 0.01, *** *p* < 0.001 vs. the control cells. (**E**) The design diagram for the establishment of intratracheal instillation of PA-infected C57BL/6J mice model. (**F**) q-PCR was employed to assess LncRNA NR_003508 expression at different time points of intratracheal instillation of PA infection (*n* = 3). ** *p* < 0.01, *** *p* < 0.001 vs. the control cells. (**G**) Subcellular localization and expression of LncRNA NR_003508 were detected by FISH probe (scale = 7.5 μm). (**H**) The proportion of LncRNA NR_003508 in the nucleus and cytoplasm detected by q-PCR in RAW264.7, using GAPDH as a cytoplasmic marker and U6 as a nuclear marker. Results are presented as mean ± SD and represent data from three independent experiments (*n* = 3 per group).

Our previous studies revealed that LncRNA NR_003508 levels increased in mouse models induced by LPS [16]. We employed q-PCR to examine if LncRNA NR_003508 plays a role in PA infection by measuring its expression at various doses and time points of PA infection of RAW264.7 and BMDM cells. As shown in Figure 2A–D, LncRNA NR_003508 expression increased with various doses and time points of PA infection and was highest at 20 MOI and 4 h. Meanwhile, the LncRNA NR_003508 expression of C57BL/6J mice infected with PA was also significantly upregulated at 6 h (Figure 2E,F). LncRNAs are known to play a role in macrophage cytoplasm by participating in ceRNA interactions [18,19]. To explore the location of LncRNA NR_003508 in macrophages, we conducted experiments involving FISH and subcellular fractionation. The findings revealed that LncRNA NR_003508 was primarily situated within the cytoplasm (Figure 2G,H) and may participate in ceRNA crosstalk.

### 3.2. LncRNA NR_003508 Provides Immune Protection Against PA Infection by Inhibiting PA Growth

**Figure 3:**
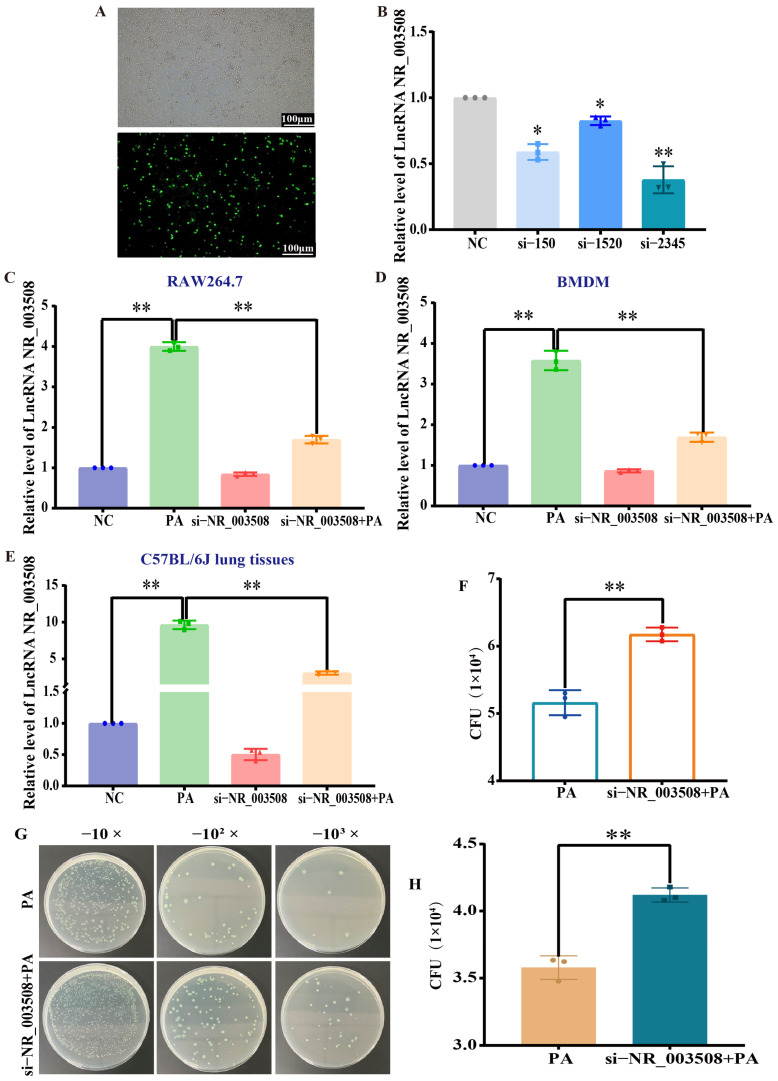
LncRNA NR_003508 provides immune protection against PA infection by inhibiting PA growth. (**A**) Light and fluorescence microscopes were used to observe the transfection efficiency of small interfering RNA (scale = 100 μm). (**B**) q-PCR detected the three LncRNA NR_003508 small interfering RNA. * *p* < 0.05, ** *p* < 0.01 vs. NC. (**C**) The expression of LncRNA NR_003508 was analyzed using q-PCR following the transfection of RAW264.7 cells with PA and si-NR_003508. ** *p* < 0.01 vs. NC, ** *p* < 0.01 vs. NC and PA. (**D**) q-PCR detected the LncRNA NR_003508 expression with PA and si-NR_003508 transfection in BMDM. ** *p* < 0.01 vs. NC, ** *p* < 0.01 vs. NC and PA. (**E**) The LncRNA NR_003508 mRNA expression with PA and si-NR_003508 transfection was detected in C57BL/6J lung tissues. ** *p* < 0.01 vs. NC, ** *p* < 0.01 vs. NC and PA. (**F**) Quantitative analysis of pulmonary bacterial load in C57BL/6J mice. ** *p* < 0.01 vs. PA. (**G**) Spread plate method detected the change in bacterial load in RAW264.7 cells with si-NR_003508 transfection. (**H**) Quantitative analysis of intracellular bacterial load. ** *p* < 0.01 vs. PA. Three independent experiments were performed as mean ± SD (*n* = 3, each group).

Since PA infection contributes to LncRNA NR_003508 expression. To further probe into the role of LncRNA NR_003508 in PA infection, we synthesized three small interfering RNAs and then selected the best knockdown effect for subsequent experiments to establish a PA infection binding LncRNA NR_003508 knockdown model (Figure 3A,B). Subsequently, we detected the knockdown effect of LncRNA NR_003508 in RAW264.7, BMDM macrophages, and mouse lung, respectively. It was found that the si-NR_003508 + PA group could significantly reduce the LncRNA NR_003508 expression compared with the PA infection group (Figure 3C–E). The results showed that the small interference could be used in subsequent research. First of all, it is unknown whether LncRNA NR_003508 is related to clearance of PA in C57BL/6J mice’s lungs. To clarify LncRNA NR_003508’s role in pulmonary bacterial load, we investigated the effect of LncRNA NR_003508 on the colonization of PA in the lung tissues of mice in vivo. As shown in Figure 3F, the PA load of si-NR_003508 + PA group was remarkably increased compared with the PA group. We further confirmed that LncRNA NR_003508 can partly promote the clearance of bacteria in macrophages (Figure 3G,H), which was consistent with the in vivo results. The findings showed that LncRNA NR_003508 conferred immune defense against PA infection by suppressing PA growth both in vivo and in vitro.

### 3.3. PA Infection Promotes Autophagy

**Figure 4:**
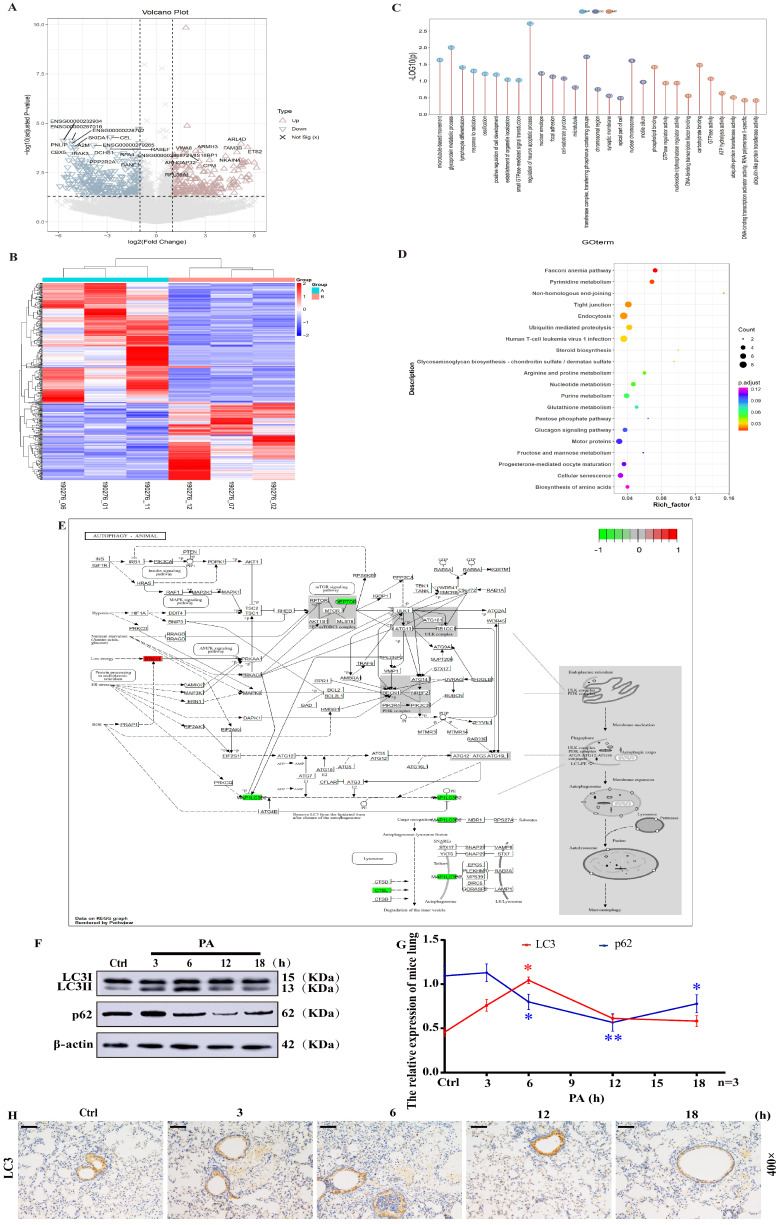
PA infection promotes autophagy. A volcano plot (**A**) and heatmap (**B**) illustrated the differentially expressed genes when comparing the PA group to the control group. Analyses of the differentially expressed genes include Gene Ontology (GO) (**C**) and Kyoto Encyclopedia of Genes and Genomes (KEGG) (**D**) studies. (**E**) The expression differences of autophagy-related genes were displayed on KEGG graph rendered by Pathview (Version 1.46.0). By superimposing the transcriptome differential expression data, the macroautophagy regulatory network with mTOR as the core switch intuitively displayed the expression trend of key autophagy genes in this experimental sample. (**F**) The expression levels of LC3 and p62 in C57BL/6J lung tissue infected with PA at different time points were assessed using Western blot analysis. (**G**) The gray value ratios of LC3II/LC3I and p62/β-actin (*n* = 3). * *p* < 0.05, ** *p* < 0.01 vs. the control cells. (**H**) IHC was used to observe the expression of LC3 in mouse lung tissue by intratracheal instillation of PA at varied time points. The tissue sections were structurally intact. After PA infection, there was marked cell recruitment (nuclei blue) and a significant increase in the number of LC3-positive cells (positive signal was mainly brown-yellow to tan) at 3 and 6 h. The data were based on at least three independent experiments presented as mean ± SD (*n* = 3, each group).

**Figure 5:**
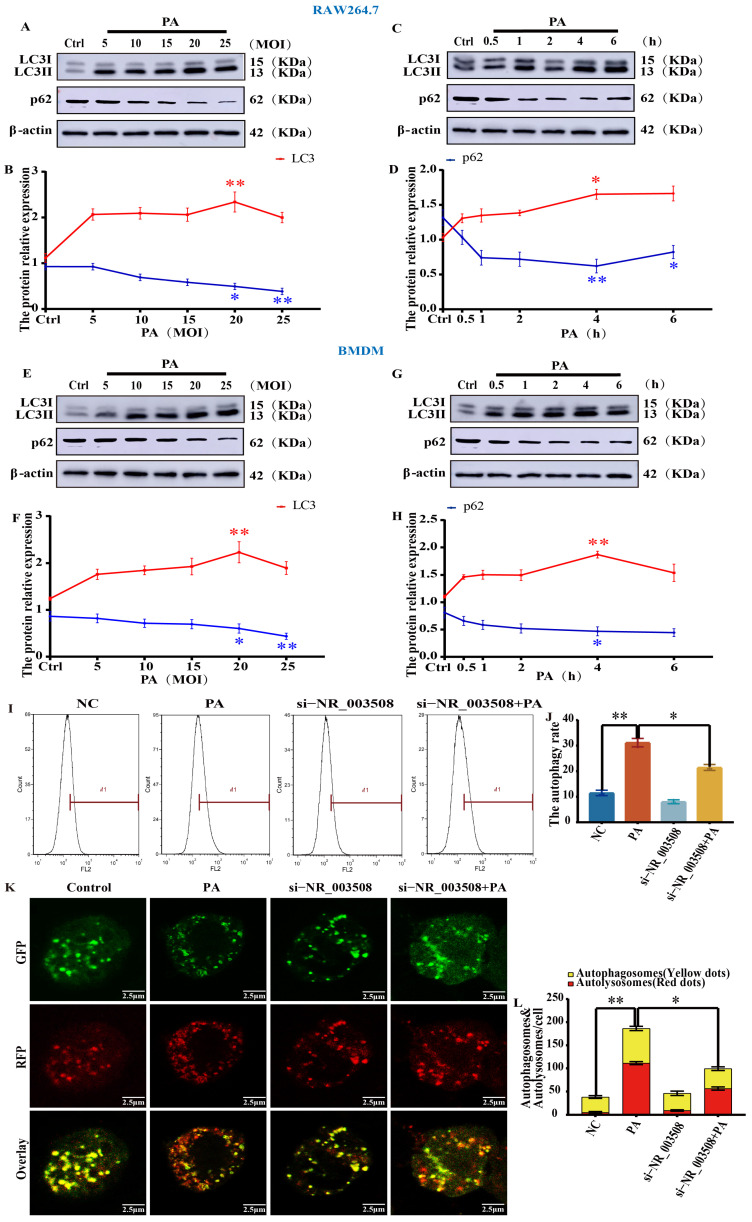
LncRNA NR_003508 accelerates autophagy with PA infection. (**A**,**E**) The LC3 and p62 expression levels with varying doses of PA infection were detected by Western blot in RAW264.7 and BMDM. (**C**,**G**) The LC3 and p62 expression infected with PA for varied time points were detected by Western blot in RAW264.7 and BMDM. (**B**,**D**,**F**,**H**) The gray value ratios of LC3II/LC3I and p62/β-actin were computed to create the line chart (*n* = 3). * *p* < 0.05, ** *p* < 0.01 vs. the control cells. (**I**) Figure of the change of autophagy rate in RAW264.7 cells following transfection with si-NR_003508 by flow cytometry. (**J**) Quantitative analysis of autophagy rate. * *p* < 0.05 vs. PA, ** *p* < 0.01 vs. NC. In (**I**), the X-axis represented the fluorescence intensity of autophagy positive markers, and the Y-axis paired the number of cells under the fluorescence intensity. The area of autophagy-positive cells was defined by M1 gate, and the left side of M1 gate was autophagy-negative cells; that is, autophagy rate = M1-positive cells ÷ total cells × 100%. (**K**) The change in autophagy flux in RAW264.7 cells following transfection with si-NR_003508 was assessed using mRFP-GFP-LC3 (scale = 2.5 μm). (**L**) Quantitative analysis of autophagy flux. * *p* < 0.05 vs. PA, ** *p* < 0.01 vs. NC. The representativeness of three independent experiments was shown as mean ± SD (*n* = 3, each group).

Undoubtedly, autophagy is crucial for the host’s defense against PA. Hence, we used the Gene Expression Omnibus (GEO) database GSE295607 to screen differentially expressed autophagy factors with PA infection. In our differential expression analysis, conducted with the training set, we identified a total of 531 transcription factors when comparing the PA group with the control group (Figure 4A,B). Of these factors, 222 were upregulated, and 309 genes were downregulated (Table S1). Further enrichment analysis of these transcription factors uncovered a total of 4521 enriched Gene Ontology (GO) terms, which included 3264 related to Biological Process (BP), 418 pertaining to Cellular Component (CC), and 569 associated with Molecular Function (MF). The top 30 terms identified encompassed ‘microtubule-based movement’, ‘glycoprotein metabolic process’, ‘cell-substrate junction’ and ‘GTPase regulator activity’. Notably, the terms related to autophagy included ‘ubiquitin-protein transferase activity’ and ‘ubiquitin-like protein transferase activity’ (Figure 4C). Additionally, KEGG analysis further emphasized the role of these differentially expressed genes (DEGs) within 236 pathways. Among them, the pathways related to autophagy include ‘Endocytosis’, ‘Ubiquitin mediated proteolysis’, and ‘Cellular senescence’ (Figure 4D). This analytical approach not only confirmed our differential expression findings but also shed light on the altered biological functions and signaling pathways implicated in PA infection. Finally, we focused on the expression differences of related factors during autophagy. Our findings indicated a pronounced correlation with LC3, which was identified as a core protein and may serve as a key insight for identifying potential therapeutic targets against PA infection. In addition to LC3 mentioned above, we also found that p62 played a key role in autophagy through a literature review [20,21,22]. So, to explore whether PA infection induces the occurrence of autophagy, Western blot was employed to assess the expression levels of LC3 and p62 in lung tissues from C57BL/6J mice. As displayed in Figure 4F–H, the LC3 expression was also significantly increased at 6 h of intratracheal instillation of PA infection, while the p62 expression was significantly reduced. Further in RAW264.7 and BMDM cells, the expression of LC3 was upregulated by the time and dose of PA with 20 MOI and 4 h, while the p62 expression was decreased (Figure 5A–H). Therefore, we speculated that PA infection promoted autophagy.

### 3.4. LncRNA NR_003508 Accelerates Autophagy with PA Infection

To verify this hypothesis, we adopted the cationic amphiphilic tracer (CAT) dye in CYTO-ID^®^ Autophagy Detection Kit to selectively label the accumulated autophagic vesicles, and the living cells were analyzed through flux cytometry. The results indicated that LncRNA NR_003508 could promote autophagy rate in RAW264.7 stimulated by PA (Figure 5I,J). The process of autophagy is dynamic, and the detection of autophagy flux is the gold standard method to accurately observe the autophagy process. And the optimal method for detecting autophagy flux. Therefore, we used mRFP-GFP-LC3 double-labeled adenovirus to infect RAW264.7 cells, and the results demonstrated that the number of yellow spots (autophagosomes) and red spots (autolysosomes) in PA-treated cells was increased compared with the control. Moreover, red spots were higher than those of yellow spots. In comparison to the PA group, the si-NR_003508 + PA group increased yellow fluorescent spots and decreased red fluorescent spots (Figure 5K,L). Results indicated that LncRNA NR_003508 promoted the autophagic flux of macrophages with PA infection, that is, facilitated the occurrence of autophagy. These findings verified that LncRNA NR_003508 played a dominant role in autophagy with PA infection.

### 3.5. LncRNA NR_003508 Contributes Autophagosome Formation with PA-Induced Autophagy

**Figure 6:**
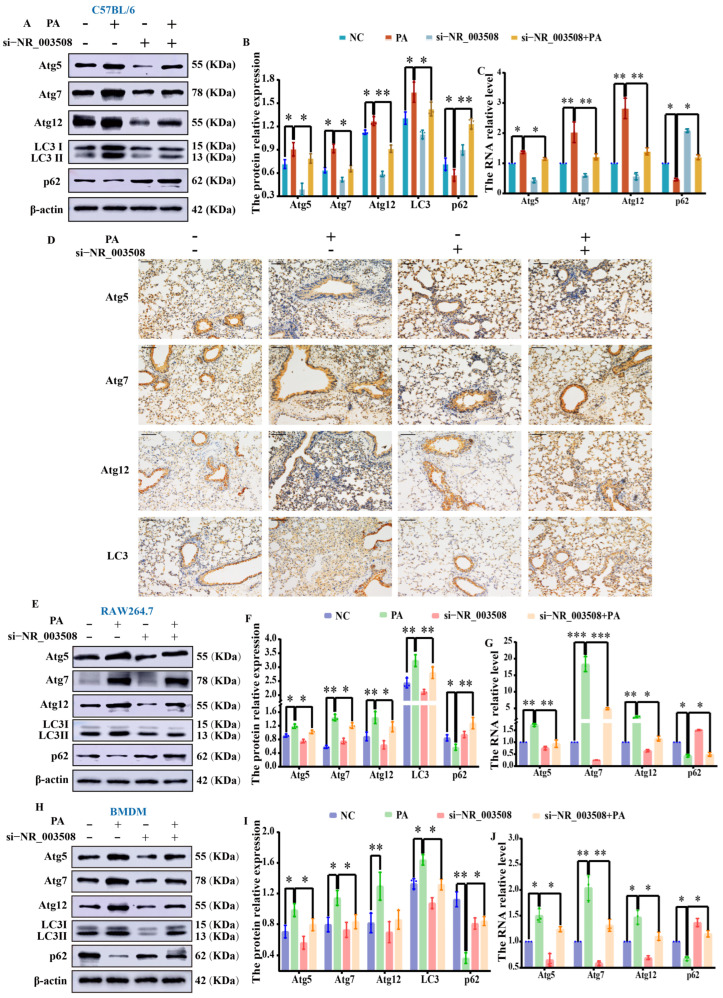
LncRNA NR_003508 promotes the relevant factors expression of autophagosome formation with PA infection. (**A**) Western blot was used to detect the expression of autophagy proteins in mouse lung tissue by intratracheal instillation of PA and si-NR_003508. (**B**) The gray value analysis results. * *p* < 0.05 vs. NC, * *p* < 0.05, ** *p* < 0.01 vs. NC and PA. (**C**) The mRNA levels of autophagy-related factors were detected by q-PCR in mouse lung tissue by si-NR_003508 transfection. * *p* < 0.05, ** *p* < 0.01 vs. NC and PA. (**D**) IHC was used to detect the levels of autophagy proteins in mouse lung tissue with si-NR_003508 transfection (400×, scale bar = 100 μm). The tissue section structure is intact. Compared with the control group, there is lymphocyte infiltration in the interstitium of the PA infection group, and the cells are significantly recruited (the cell nuclei are blue), while the number of antibody-positive cells significantly increases (yellow to brown) and the positive cells significantly decrease in the si-NR_003508 + PA group. The expression of Atg5, Atg7, Atg12, LC3, and p62 in RAW264.7 (**E**) and BMDM (**H**) was detected by Western blot with LncRNA NR_003508 knockdown. The gray scale analysis results in RAW264.7 (**F**) and BMDM (**I**). * *p* < 0.05 vs. NC and PA, ** *p* < 0.01 vs. NC and PA. q-PCR was used to detect the expression of Atg5, Atg7, Atg12, and p62 in RAW264.7 (**G**) and BMDM (**J**) with LncRNA NR_003508 knockdown. * *p* < 0.05 vs. PA, ** *p* < 0.01 vs. NC and PA, *** *p* < 0.001 vs. NC and PA. Three independent experiments were performed as mean ± SD (*n* = 3, each group).

Autophagy was orchestrated by a suite of genes associated with autophagy, including mTOR, Beclin1, LC3, and Atg5, which controlled different stages of autophagy (initiation, progression, and maturation) [23]. Accumulating evidence indicates that ATG7, ATG12, and ATG5 drive the elongation of autophagosomal membranes via the ubiquitin-like conjugation system, thereby contributing to autophagosome biogenesis. As a structural protein of autophagosomes, LC3 not only mediated membrane elongation and autophagosome closure but also directed autophagosomes to target lysosomes [24]. Concurrently, p62 acted as a scaffold bridging LC3 and polyubiquitinated proteins, selectively sequestering them into autophagosomes and subsequently degrading them by proteolytic enzymes within autolysosomes during autophagosome formation [25]. Notably, p62 expression exhibited a negative correlation with autophagic activity. To elucidate the mechanism of LncRNA NR_003508 in autophagy, we examined the specific stage of autophagy influenced by LncRNA NR_003508. First, we detected the levels of autophagy-related factors using Western blot, immunohistochemistry, and q-PCR. It was found that compared to the control, Atg5, Atg7, Atg12, and LC3 expression were markedly elevated following PA infection. Simultaneously, in the si-NR_003508 + PA group, the levels of Atg5, Atg7, Atg12, and LC3 were significantly reduced in comparison to the PA group, while p62 expression was found to be increased (Figure 6A–D). Furthermore, we obtained the same results in RAW264.7 and BMDM macrophages (Figure 6E–J).

**Figure 7:**
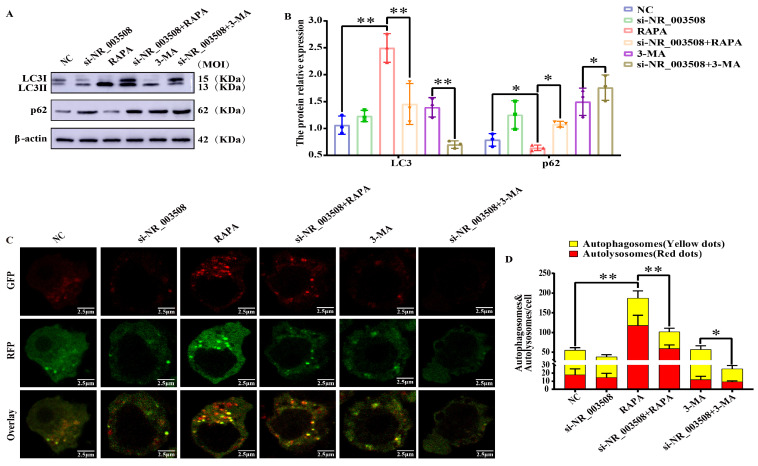
LncRNA NR_003508 boosts autophagosome formation. (**A**) The expression of LC3 and p62 in RAW264.7 was detected by Western blot transfecting with si-NR_003508, RAPA, and 3-MA. (**B**) The gray scale analysis results. * *p* < 0.05, ** *p* < 0.01 vs. si-NR_003508. (**C**) The changes in the tandem mRFP-GFP-LC3 punctas were observed by confocal microscope (scale = 2.5 μm). (**D**) Quantitative analysis of yellow and red fluorescent spots. * *p* < 0.05, ** *p* < 0.01 vs. si-NR_003508. Results of three independent experiments were shown as mean ± SD (*n* = 3, each group).

To enhance the persuasiveness of the results, we used the agonist (RAPA) and inhibitor (3-MA) to activate and inhibit autophagosome formation, respectively, to distinguish between increased autophagosome formation and altered downstream turnover. Compared with the NC group, RAPA significantly enhanced autophagy with elevated LC3 expression and decreased p62 expression (Figure 7A,B) and activated autophagic flux (Figure 7C,D), while si-NR_003508 directly inhibited RAPA-induced autophagy. Furthermore, si-NR_003508 remarkably aggravated the 3-MA-inhibiting autophagy effect. It indicated that LncRNA NR_003508 directly promoted autophagosome formation rather than downstream turnover.

### 3.6. LncRNA NR_003508 Targets Autophagy-Related miRNA

**Figure 8:**
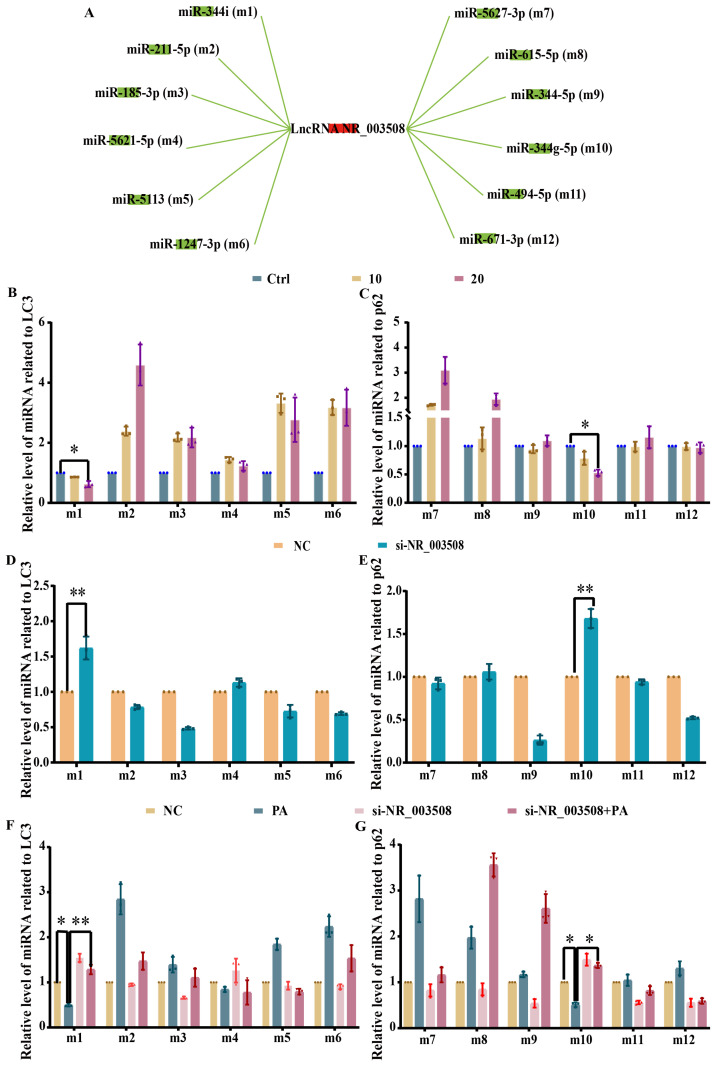
LncRNA NR_003508 targets autophagy-related miRNA. (**A**) Prediction indicator of the interaction between LncRNA NR_003508 and autophagy proteins LC3 and p62-related miRNA (left are associated with LC3, right are associated with p62). The expression of LC3 (**B**) and p62 (**C**) miRNA was detected by q-PCR in different MOIs of PA infection. * *p* < 0.05 vs. Control. q-PCR was used to detect the expression of LC3 (**D**) and p62 (**E**) miRNA with LncRNA NR_003508 interference. ** *p* < 0.01 vs. NC. The LC3 (**F**) and p62 (**G**) expression with PA infection and LncRNA NR_003508 interference was detected by q-PCR. * *p* < 0.05 vs. NC and PA, ** *p* < 0.01 vs. PA. Three independent experiments were shown as mean  ±  SD (*n* = 3).

Of note, our previous results have confirmed that LncRNA NR_003508 is mainly located in the cytoplasm and functions as ceRNAs. Generally speaking, an important regulatory pattern of lncRNAs is to act as ceRNAs, allowing them to modulate gene expression by binding to miRNA in a competitive manner [26]. Therefore, we used multiple bioinformatics databases to screen 12 miRNAs conditional on targeting LncRNA NR_003508 and targeting the autophagy key proteins LC3 and p62, respectively (Figure 8A). As illustrated in Figure 8B–E, out of the 12 miRNAs analyzed, miR-344i and miR-344g-5p expression were decreased at 20 MOI of PA infection compared to the control or NC group. In contrast, the application of si-NR_003508 significantly raised the levels of miR-344i and miR-344g-5p. Further analysis with si-NR_003508 in conjunction with PA infection revealed an increase in the expression of miR-344i and miR-344g-5p due to PA infection. However, si-NR_003508 suppressed the expression of miR-344i and miR-344g-5p. (Figure 8F,G). Therefore, miR-344i and miR-344g-5p were selected for subsequent research.

### 3.7. LncRNA NR_003508 Sponges miR-344i to Target LC3 and Stimulates the Conversion of LC3-I to LC3-II

**Figure 9:**
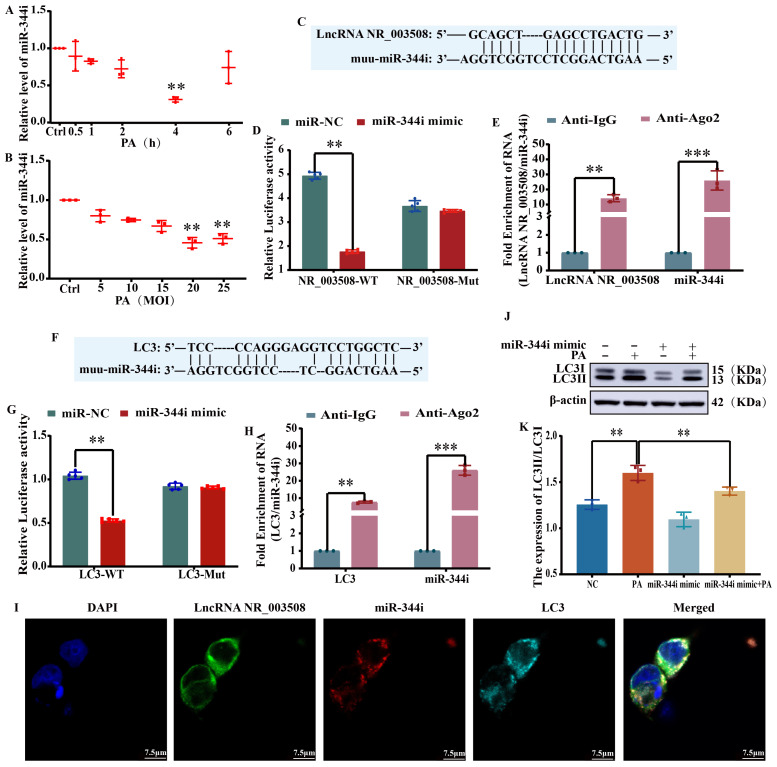
LncRNA NR_003508 sponges miR-344i to target LC3 and stimulates the conversion of LC3I to LC3II. (**A**,**B**) q-PCR was used to detect miR-344i levels at different times and MOIs of PA infection. ** *p* < 0.01 vs. Control. (**C**) Bielefeld Bioinformatics Service identified the binding sites for LncRNA NR_003508 and miR-344i. (**D**) The luciferase reporter assay confirmed the interaction between LncRNA NR_003508 and miR-344i (*n* = 3). ** *p* < 0.01 vs. the NC group. (**E**) RIP verified the connection between LncRNA NR_003508 and miR-344i. ** *p* < 0.01, *** *p* < 0.001 vs. the IgG group. (**F**) RNA22 V2 prediction software was used for bioinformatics analysis. (**G**) The interaction between miR-344i and LC3 was validated by a dual-luciferase reporter assay. ** *p* < 0.01 vs. the NC group. (**H**) RIP experiment verified the relationship between miR-344i and LC3. ** *p* < 0.01, *** *p* < 0.001 vs. the IgG group. (**I**) Immunofluorescence was utilized to observe the co-localization of LncRNA NR_003508, miR-344i, and LC3. (scale bar = 7.5 μm). (**J**) The conversion of LC3I to LC3II with miR-344i mimic transfection was detected by Western blot. (**K**) The gray value analysis results. ** *p* < 0.01 vs. the NC and PA group. The results are presented as mean ± SD from three separate experiments (*n* = 3, each group).

The expression of miR-344i was first detected at different times and doses of PA infection. And the results were shown in Figure 9A,B, miR-344i expression was significantly reduced at 4 h and 20 MOI. Thus, we produced the miR-344i mimic for more detailed analysis. The predicted interaction location between LncRNA NR_003508 and miR-344i was anticipated with BiBiServ-RNAhybrid. (Figure 9C). Then, we found that the miR-344i mimic significantly reduced the luciferase activity of LncRNA NR_003508-WT but not LncRNA NR_003508-Mut (Figure 9D). In addition, we verified the physical site between LncRNA NR_003508 and miR-344i using the RNA immunoprecipitation (RIP) assay (Figure 9E). In this respect, LncRNA NR_003508 functions as a ceRNA to trap miR-344i. Furthermore, we analyzed the interaction sites between miR-344i and target mRNA LC3 using RNA22 V2 prediction software (version, java1.6) (Figure 9F). As shown in Figure 9G, the luciferase reporter assay verified the predicted mutual binding interaction, with the miR-344i mimic significantly lowering the relative luciferase activity of LC3-WT, while LC3-MUT remained with no significant difference, indicating that LC3 may be a direct target gene of miR-344i. Within the RNA-induced silencing complex (RISC), Argonaute (Ago) serves as the catalytic component [27]. Then, the RIP experiment revealed that anti-Ago2 could significantly deposit LC3 and miR-344i (Figure 9H). Immunofluorescence results further confirmed the co-localization of LncRNA NR_003508, miR-344i, and LC3 (Figure 9I). To further explore the effect of the miR-344i mimic on the conversion of LC3-I to LC3-II. A Western blot was used to find that the conversion of LC3I to LC3II was increased in the PA infection group while decreased in the miR-344i mimic + PA group (Figure 9J,K). In conclusion, LncRNA NR_003508 can sponge miR-344i to relieve the silence of LC3 and promote the conversion of LC3I to LC3II, thereby facilitating the occurrence of autophagy.

### 3.8. LncRNA NR_003508 Targets p62 by Sponging miR-344g-5p

**Figure 10:**
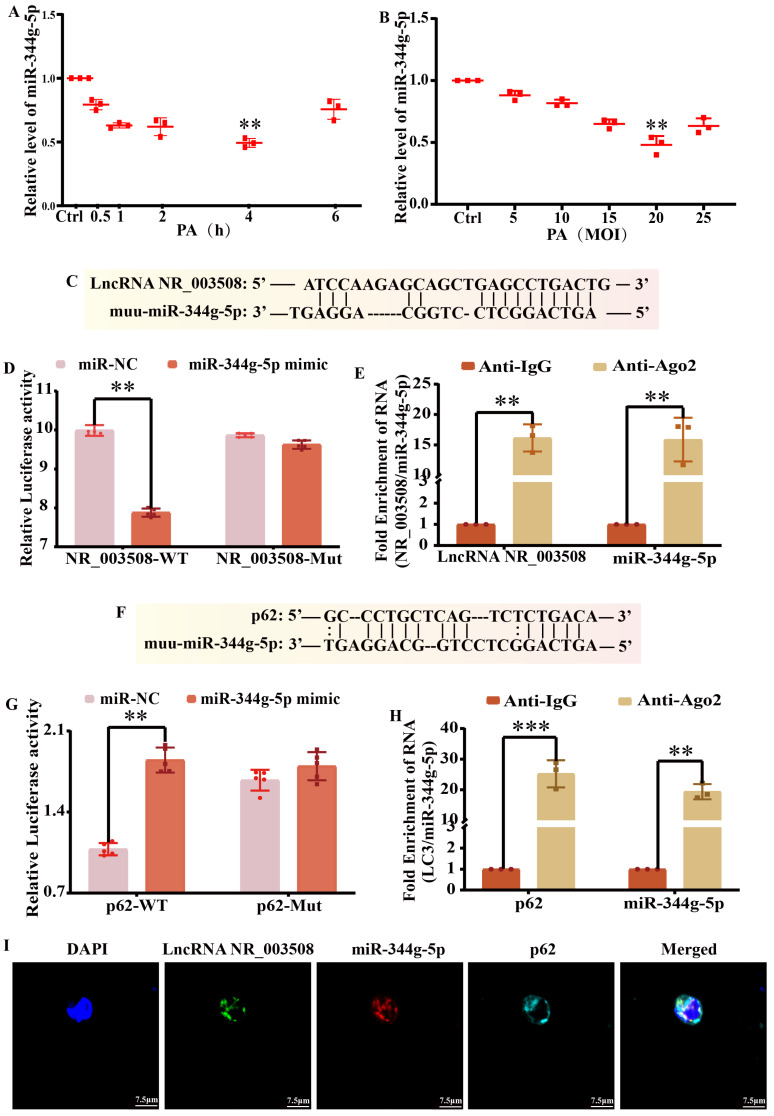
LncRNA NR_003508 targets p62 by sponging miR-344g-5p. (**A**,**B**). The miR-344g-5p expression at different times and MOIs of PA infection was detected by q-PCR. ** *p* < 0.01 vs. Control. (**C**) Bielefeld Bioinformatics Service was used to forecast the binding sites between LncRNA NR_003508 and miR-344g-5p. (**D**) The interaction between LncRNA NR_003508 and miR-344g-5p was confirmed using a luciferase reporter (*n* = 3). ** *p* < 0.01 vs. the NC group. (**E**) RIP experiment verified the relationship between LncRNA NR_003508 and miR-344g-5p. ** *p* < 0.01, ** *p* < 0.01 vs. the IgG group. (**F**) Bioinformatics analysis by RNA22 V2. (**G**) The interaction between miR-344g-5p and p62 was validated by luciferase reporter. ** *p* < 0.01 vs. the NC group. (**H**) RIP confirmed the relationship between miR-344g-5p and p62. *** *p* < 0.001, ** *p* < 0.01 vs. the IgG group. (**I**) Immunofluorescence revealed the co-localization of LncRNA NR_003508, miR-344g-5p, and p62 (scale bar = 7.5 μm). The results from three separate experiments are presented as mean ± SD (*n* = 3, each group).

**Figure 11:**
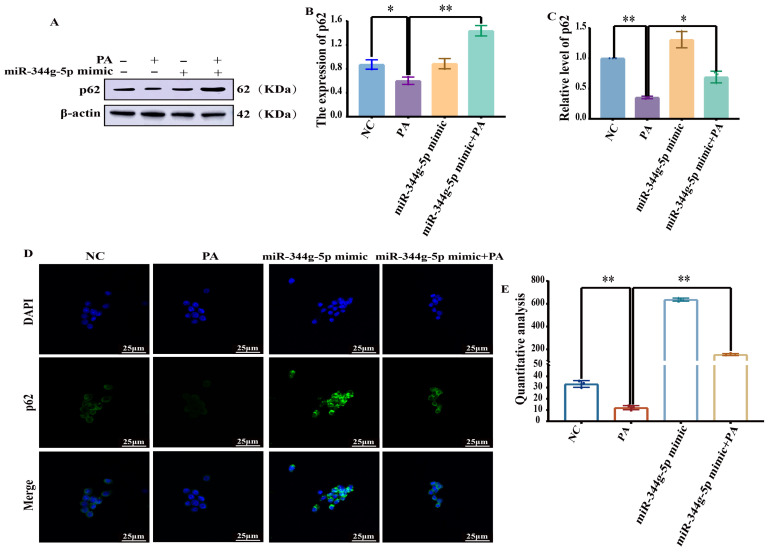
LncRNA NR_003508 targets p62 by sponging miR-344g-5p. (**A**) Western blot was employed to measure p62 expression following transfection with the miR-344g-5p mimic. (**B**) The gray value analysis results. * *p* < 0.05 vs. NC. ** *p* < 0.01 vs. PA. (**C**) q-PCR was utilized to assess p62 expression with miR-344g-5p mimic transfection. ** *p* < 0.05 vs. NC, * *p* < 0.05 vs. PA. (**D**) Immunofluorescence was used to observe the p62 expression with miR-344g-5p mimic transfection (scale bar = 25 μm). (**E**) Quantitative analysis of Immunofluorescence. ** *p* < 0.01 vs. NC and PA. The data were based on at least three independent experiments (*n* = 3, each group).

Next, we examined the expression of miR-344g-5p with PA infection at different times and doses. At 4 h and 20 MOI, there was a significant decrease in miR-344g-5p expression (Figure 10A,B). Therefore, the miR-344g-5p mimic was synthesized for further research. To clarify how LncRNA NR_003508 interacts with miR-344g-5p, we employed BiBiServ-RNAhybrid software (version, 2004) to predict their interaction sites. The specific interaction sites were shown in Figure 10C, from which we can speculate that miR-344g-5p may be another target miRNA of LncRNA NR_003508. Afterward, the luciferase reporter assay demonstrated that introducing the miR-344g-5p mimic led to a significant decrease in the luciferase activity of LncRNA NR_003508-WT, but not LncRNA NR_003508-Mut (Figure 10D). The RIP assay showed that both LncRNA NR_003508 and miR-344g-5p were found to be enriched in the compound precipitated by anti-Ago2, revealing that LncRNA NR_003508 sponged miR-344g-5p (Figure 10E). Moreover, we used RNA22 V2 to analyze the interaction sites between miR-344g-5p and the target mRNA p62. As shown in Figure 10F, the 3′-UTR region of p62 included the binding sites for miR-344g-5p, speculating p62 was the candidate target of miR-344g-5p. After transfecting the miR-344g-5p mimic, the luciferase activity was increased in the LncRNA NR_003508-WT group but not in the LncRNA NR_003508-Mut (Figure 10G), indicating that miR-344g-5p can promote p62 expression. RIP verified that miR-344g-5p directly targets p62. (Figure 10H). Furthermore, immunofluorescence results confirmed the co-localization of LncRNA NR_003508, miR-344g-5p, and p62 (Figure 10I). The impact of the miR-344g-5p mimic on p62 expression was examined using Western blot and q-PCR. As shown in Figure 11A–C, PA infection reduced p62 expression, which is consistent with our previous experiments. In comparison to the PA group, the miR-344g-5p mimic + PA group showed a significant increase in p62 expression. The immunofluorescence displayed the same results (Figure 11D,E). Combined with the above results, we concluded that LncRNA NR_003508 degraded p62 by binding to miR-344g-5p, thereby promoting macrophage autophagy.

## 4. Discussion

Growing evidence indicates that LncRNAs assume critical regulatory functions across various biological processes [28]. More importantly, LncRNA H19 promoted chondrocyte autophagy by targeting miR-148a in cases of post-traumatic osteoarthritis, indicating the role of LncRNAs in autophagic mechanisms [29]. In this study, we observed a notable increase of LncRNA NR_003508 expression in both macrophages and C57BL/6J mice with *Pseudomonas aeruginosa* (PA) infection, suggesting that LncRNA NR_003508 may play a core role in immune response during PA infection. Given that the functional roles of LncRNAs are closely associated with their subcellular localization [30], we further investigated the cellular distribution of LncRNA NR_003508 in RAW264.7 cells utilizing FISH and nuclear-cytoplasmic separation assays. Our results demonstrated that LncRNA NR_003508 was expressed in both the nucleus and cytoplasm following PA infection, with predominant expression observed in the cytoplasm. These findings implied that LncRNA NR_003508 may participate in ceRNA-mediated regulatory networks.

Our previous findings confirmed that LncRNA NR_003508 expression is significantly upregulated with PA infection. RNA interference (RNAi) technology, a widely utilized method for gene silencing, enables efficient knockdown of target gene expression in cultured cells and mice [31]. To further investigate the functional role of LncRNA NR_003508, we designed and synthesized specific siRNAs targeting LncRNA NR_003508, thereby establishing the foundation for subsequent studies on its regulatory mechanisms in PA-induced immune responses. PA infection activated the host innate immune response, among which autophagy was a critical mechanism for pathogen clearance and represented a key immune defense strategy against PA infection. Our findings indicated that LncRNA NR_003508 contributed to the elimination of intracellular PA. To determine whether this clearance was associated with cellular autophagy, we further used the Gene Expression Omnibus (GEO) database to screen differentially expressed autophagy factors with PA infection and found that autophagy factors showed a stronger correlation. Then, we conducted experimental validation. Monitoring autophagic flux using mRFP-GFP-LC3 adenovirus is widely regarded as the “gold standard” for assessing autophagy flux. Using this method, we found that LncRNA NR_003508 enhances autophagic flux in macrophages with PA infection. Additionally, LncRNA NR_003508 was shown to increase the autophagy rate in macrophages. It was demonstrated that LncRNA NR_003508 promoted the occurrence of autophagy in cells, thereby partly eliminating intracellular pathogenic bacteria. Autophagy was regulated by a cascade of autophagy-related genes [32,33]. Recent studies showed that non-coding RNAs mainly regulate the expression of ATG genes, thereby regulating the autophagy process [5]. At the molecular level, we observed that LncRNA NR_003508 promoted the expression of Atg5, Atg7, Atg12, and LC3, while p62 was the opposite. The results indicated that LncRNA NR_003508 stimulated the formation of autophagosomes, thereby partly facilitating the clearance of intracellular pathogens.

Emerging evidence suggested that cytoplasmic LncRNAs can regulate the expression of downstream target genes by competitively absorbing microRNAs (miRNAs). By functioning as a ceRNA, lncRNA muscle growth regulator (lncMGR) competitively sequestered miR-2131-5p, thus safeguarding MYH1A from miR-2131-5p-induced degradation [34]. For autophagy, research has verified that the metastasis-associated lung adenocarcinoma transcript 1 (MALAT1)-miR-30c-5p-CTGF/ATG5 axis triggers experimental silicosis [35]. Therefore, we first searched for autophagy-related miRNAs potentially interacting with LncRNA NR_003508 using bioinformatics databases. Subsequently, we screened for matching miRNAs and analyzed the interaction sites between LncRNA NR_003508 and miRNA. Luciferase reporter assays and co-localization experiments further confirmed the interaction between LncRNA NR_003508 and miR-344i and miR-344g-5p, indicating that LncRNA NR_003508 is sponging miR-344i and miR-344g-5p. Moreover, microRNAs are approximately 22 nucleotides in length and can either silence mRNA expression by binding to the 3′-UTR region or amplify target gene expression [36,37]. We found that miR-344i and miR-344g-5p regulated the expression of LC3 and p62 by binding to their respective 3′-UTR regions, which was consistent with the above results. The LncRNA/miRNA/mRNA regulatory network formed through interactions among LncRNAs, miRNAs, and mRNAs plays a critical role in the progression of infectious diseases by modulating cellular processes such as autophagy, apoptosis, and necrosis. A notable example is that in hepatocellular carcinoma, LncRNA myocardial infarction-associated transcript (MIAT) influenced autophagy and apoptosis in macrophages infected with *M.tb* via the miR-665/ULK1 pathway [38]. In our study, we also verified that LncRNA NR_003508 acts as a sponge for miRNA, relieving its inhibitory effect on LC3 and promoting the conversion of LC3I to LC3II. Additionally, LncRNA NR_003508 can sponge miR-344g-5p to facilitate p62 degradation, thereby enhancing macrophages autophagy with PA infection.

PA has been shown to induce cellular autophagy, a process that is crucial for the host in eliminating pathogenic bacteria. In this study, we demonstrated that the LncRNA NR_003508 can enhance autophagy in macrophages with PA infection through miR-344i/LC3 and miR-344g-5p/p62 axes, partly leading to effective bacterial clearance. Notably, targeted therapy for LncRNAs has emerged as one of the most extensively investigated and promising strategies in the field of disease treatment. LncRNA NBR2, a key regulator of cancer metabolism, promoted Beclin1-dependent autophagy in hepatocellular carcinoma through the ERK and JNK pathways, regulating the proliferation of hepatocellular carcinoma [39]. These findings suggested that LncRNA NBR2 served as a potential biomarker in cancer therapy. Additionally, LncRNA PVT1 is identified as a therapeutic target in clinical tumorigenesis [40]. Moreover, the development of resistance to different chemotherapy drugs in cancer is also related to lncRNA-mediated autophagy. Specifically, the suppression of XIST decreased paclitaxel resistance in breast cancer cells by influencing hsa-let-7d-5p/ATG16L1 and controlling autophagy [41]. Nonetheless, there are certain limitations to this study. We have not yet fully demonstrated that the observed effect on bacterial clearance is specifically mediated through autophagy. si-NR_003508 increases bacterial load in vitro and in vivo, but macrophages can influence bacterial killing through many pathways beyond autophagy. Therefore, what role other modes of death play in this process, and whether autophagy plays a central role, need to be further explored.

In summary, *Pseudomonas aeruginosa* infection increases LncRNA NR_003508 expression in vivo and in vitro, which facilitates the conversion of LC3I to LC3II, a critical step in autophagosome formation, by targeting miR-344i. Simultaneously, it facilitated p62 degradation by binding to miR-344g-5p, thereby further promoting autophagy and partly boosting the clearance of *Pseudomonas aeruginosa*. Our findings imply that LncRNA NR_003508 might offer theoretical and experimental insights into the mechanisms behind *Pseudomonas aeruginosa* infection.

## 5. Conclusions

This study identifies LncRNA NR_003508 as a functional mediator of *Pseudomonas aeruginosa* infection in autophagosome formation and demonstrates that it directly promoted autophagosome formation rather than downstream turnover. More importantly, LncRNA NR_003508 enhances the formation of autophagosomes induced by PA infection via the miR-344i/LC3 and miR-344g-5p/p62 axes, partly boosting the clearance of PA. These findings provide a theoretical basis and experimental support for understanding the mechanisms underlying PA infection.

## Figures and Tables

**Table 1:** The primers sequence.

Primer	Sequence (5′-3′)
LncRNA NR_003508	F: GTATGAGGAGAAGGTGCGGC
	R: CCAGAACTCTGGTCCCCAAT
Atg5	F: CACCCCTGAAATGGCATTATCC
	R: TGGACAGTGTAGAAGGTCCTTT
Atg7	F: TCTGGGAAGCCATAAAGTCAGG
	R: GCGAAGGTCAGGAGCAGAA
Atg12	F: TGCTGAAGGCTGTAGGAGAC
	R: GAGGCCACCAGTTTAAGGAA
p62	F: CGCAGAACAGAGTTACGAAGG
	R: TCCCATTCCAGTCATCTTGTC
β-actin	F: TGAGAGGGAAATCGTGCGTGACAT
	R: ACCGCTCGTTGCCAATAGTGATGA
miR-344i	RT: CTCAACTGGTGTCGTGGAGTCGGCAATTCAGTTGAGTCCAGCCA
	F: ACACTCCAGCTGGCAAGTCAGGCTCCTGGCTG
miR-5113	RT: CTCAACTGGTGTCGTGGAGTCGGCAATTCAGTTGAGACAGGATC
	F: ACACTCCAGCTGGCACAGAGGAGGAGAGAGAT
miR-5621-5p	RT: CTCAACTGGTGTCGTGGAGTCGGCAATTCAGTTGAGTCAGGGCG
	F: ACACTCCAGCTGGCAGGAGGTCCTGGGGCCGC
miR-1247-3p	RT: CTCAACTGGTGTCGTGGAGTCGGCAATTCAGTTGAGGCTCCAGT
	F: ACACTCCAGCTGGCCGGGAACGTCGAGACTGG
miR-211-5p	RT: CTCAACTGGTGTCGTGGAGTCGGCAATTCAGTTGAGAGGCAAAG
	F: ACACTCCAGCTGGCTTCCCTTTGTCATCCTTT
miR-185-3p	RT: CTCAACTGGTGTCGTGGAGTCGGCAATTCAGTTGAGACCAGAGG
	F: ACACTCCAGCTGGCAGGGGCTGGCTTTCCTCT
miR-5627-3p	RT: CTCAACTGGTGTCGTGGAGTCGGCAATTCAGTTGAGCGCAGGGC
	F: ACACTCCAGCTGGCAGAGGGTGCGCCGGGCCCT
miR-615-5p	RT: CTCAACTGGTGTCGTGGAGTCGGCAATTCAGTTGAGCTCCAGCC
	F: ACACTCCAGCTGGCAGGAAGCCCTGGAGGGGCT
miR-344-5p	RT: CTCAACTGGTGTCGTGGAGTCGGCAATTCAGTTGAGCCTGGAAT
	F: ACACTCCAGCTGGCAGTCAGGCTCCTGGCTAGA
miR-344g-5p	RT: CTCAACTGGTGTCGTGGAGTCGGCAATTCAGTTGAGACTCCTGC
	F: ACACTCCAGCTGGCAGTCAGGCTCCTGGCAGGA
miR-494-5p	RT: CTCAACTGGTGTCGTGGAGTCGGCAATTCAGTTGAGGAGAAGAC
	F: ACACTCCAGCTGGCAGGTTGTCCGTGTTGTCTT
miR-671-3p	RT: CTCAACTGGTGTCGTGGAGTCGGCAATTCAGTTGAGCTCCAGCC
	F: ACACTCCAGCTGGCAGGAAGCCCTGGAGGGGCT
miRNA Universal Reverse sequence	TGGTGTCGTGGAGTCG
SnoRNA202	RT: CTCAACTGGTGTCGTGGAGTCGGCAATTCAGTTGAGCCTGGAAT
	F: ACACTCCAGCTGGCAGTCAGGCTCCTGGCTAGA

**Table 2:** The FISH fluorescent probes sequence.

Probe #	Probe (5′->3′)	Probe Position	Percent GC (%)
1	ccacagaatcctgtgcaata	33	45.0
2	tgtgttttcttctgtgctaa	129	35.0
3	tccactgggtaagttgactg	159	50.0
4	agattgttcacagactcctg	203	45.0
5	agtcgatgaggtcaatgcag	253	50.0
6	cttctagtacagagctcttc	346	45.0
7	attcagcttcctcaatttca	426	35.0
8	tccacttcgatgtcatcata	476	40.0
9	cacctctgaaggatcagaga	498	50.0
10	ctgacatccaggctaatgag	581	50.0
11	agtcaggtctgggacatttg	606	50.0
12	taattccaggcaggtcaatg	628	45.0
13	gtatgtcttgatgagtctct	696	40.0
14	ttgatggtctcctgtttttg	719	40.0
15	tggtggcaatgtccacattg	757	50.0
16	agatcgggcttggtcaagat	833	50.0
17	cacgaccttgtcttcagtac	864	50.0
18	ttcttcagatggtacaccag	899	45.0
19	gcacttgactatcatgtagc	921	45.0
20	ctcattctgaagagcctcag	978	50.0
21	gatgtgtgagatgagctctg	1086	50.0
22	acttgcactctggtgacttt	1143	45.0
23	cttgtactagggcagtgatg	1255	50.0
24	ttttcggagcctggtgaata	1308	45.0
25	ccttactccaggacaagaac	1330	50.0
26	gggttttgatttgtcttctt	1475	35.0
27	atttctatagctggctcttc	1497	40.0
28	aatttcagtgaccgtgtgca	1519	45.0
29	acacttgtaaaggcagctcg	1542	50.0
30	gtagttctgtggaggttata	1587	40.0
31	gatgtcttcaagtttggact	1609	40.0
32	ttgaagtggagtcggatcga	1659	50.0
33	tgagaggaactggaagtgcc	1776	55.0
34	taggcattcagatgctggaa	1833	45.0
35	gatgatcaagggaacgtggc	1882	55.0
36	gctcagcaaacattttcagg	1913	45.0
37	ttcaggaacttcctcttctc	2013	45.0
38	cttaaccagggaatttggct	2072	45.0
39	aacacagggaaggacagagc	2095	55.0
40	ttctgtccctgaatcacaat	2121	40.0
41	atagctaccaggaaaggcag	2147	50.0
42	tcagagcataaaggctggtg	2169	50.0
43	caaatctgagtgctagcact	2191	45.0
44	ctcagagtgagcagaaagct	2215	50.0
45	atctacttgctgagctttga	2256	40.0
46	gtgaagccgttgtcttatta	2297	40.0
47	gtgggcaagaagagtactca	2319	50.0
48	aaggaatgcaacaggcagct	2383	50.0

## Data Availability

The original data presented in this study are openly available in [Gene Expression Omnibus (GEO) database GSE295607] at [https://doi.org/10.1101/2025.05.28.656674], reference number [https://pubmed.ncbi.nlm.nih.gov/40501816/, accessed on 19 July 2026]. The additional Data will be made available on request.

## Supplementary Materials

The following supporting information can be downloaded at: https://www.mdpi.com/article/10.3390/cells15141306/s1, Table S1: The related factors with PA infection; Supplementary File S2: The sequences of the LncRNA NR_003508, LC3 and p62 dual luciferase plasmids.

## Abbreviations

The following abbreviations are used in this manuscript:

PA	*Pseudomonas aeruginosa*
LncRNA	Long non-coding RNAs
FISH	Fluorescence in situ hybridisation
miRNAs	microRNAs
3′-UTR	3′-untranslated region
LPS	lipopolysaccharide
ceRNAs	competing endogenous RNAs
Ago	Argonaute
siRNA	Small Interfering RNA
NC	negative controls
HE	Hematoxylin and eosin
