# Supplementary material for: LncRNA NR_003508 Boosts Pseudomonas aeruginosa-Induced Autophagy by Facilitating the Conversion of LC3-I to LC3-II and p62 Degradation
Source: Cells. 2026 Jul 21;15(14):1306. doi: 10.3390/cells15141306 (PMC13406791; doi:10.3390/cells15141306)
Supplement: Supplementary file 1 [file cells-15-01306-s001.zip › Supplementary File 2.pdf]

The sequences of the LncRNA NR_003508, LC3 and p62 dual luciferase plasmids

1. LncRNA NR_003508 WT-hRluc-Fluc

AGATCTGCGCAGCACCATGGCCTGAAATAACCTCTGAAAGAGGAACTTGGTTAGGTACCTTCTG

AGGCGGAAAGAACCAGCTGTGGAATGTGTGTCAGTTAGGGTGTGGAAAGTCCCCAGGCTCCCC

AGCAGGCAGAAGTATGCAAAGCATGCATCTCAATTAGTCAGCAACCAGGTGTGGAAAGTCCCC

AGGCTCCCCAGCAGGCAGAAGTATGCAAAGCATGCATCTCAATTAGTCAGCAACCATAGTCCCG

CCCCTAACTCCGCCCATCCCGCCCCTAACTCCGCCCAGTTCCGCCCATTCTCCGCCCCATGGCTG

ACTAATTTTTTTTATTTATGCAGAGGCCGAGGCCGCCTCGGCCTCTGAGCTATTCCAGAAGTAGTG

AGGAGGCTTTTTTGGAGGCCTAGGCTTTTGCAAAAAGCTTGATTCTTCTGACACAACAGTCTCGA

ACTTAAGCTGCAGAAGTTGGTCGTGAGGCACTGGGCAGGTAAGTATCAAGGTTACAAGACAGG

TTTAAGGAGACCAATAGAAACTGGGCTTGTCGAGACAGAGAAGACTCTTGCGTTTCTGATAGGC

ACCTATTGGTCTTACTGACATCCACTTTGCCTTTCTCTCCACAGGTGTCCACTCCCAGTTCAATTA

CAGCTCTTAAGGCTAGAGTACTTAATACGACTCACTATAGGCTAGCCACCATGGCTTCCAAGGTG

TACGACCCCGAGCAACGCAAACGCATGATCACTGGGCCTCAGTGGTGGGCTCGCTGCAAGCAA

ATGAACGTGCTGGACTCCTTCATCAACTACTATGATTCCGAGAAGCACGCCGAGAACGCCGTGA

TTTTTCTGCATGGTAACGCTGCCTCCAGCTACCTGTGGAGGCACGTCGTGCCTCACATCGAGCCC

GTGGCTAGATGCATCATCCCTGATCTGATCGGAATGGGTAAGTCCGGCAAGAGCGGGAATGGCT

CATATCGCCTCCTGGATCACTACAAGTACCTCACCGCTTGGTTCGAGCTGCTGAACCTTCCAAAG

AAAATCATCTTTGTGGGCCACGACTGGGGGGCTTGTCTGGCCTTTCACTACTCCTACGAGCACCA

AGACAAGATCAAGGCCATCGTCCATGCTGAGAGTGTCGTGGACGTGATCGAGTCCTGGGACGA

GTGGCCTGACATCGAGGAGGATATCGCCCTGATCAAGAGCGAAGAGGGCGAGAAAATGGTGCT

TGAGAATAACTTCTTCGTCGAGACCATGCTCCCAAGCAAGATCATGCGGAAACTGGAGCCTGAG

GAGTTCGCTGCCTACCTGGAGCCATTCAAGGAGAAGGGCGAGGTTAGACGGCCTACCCTCTCCT

GGCCTCGCGAGATCCCTCTCGTTAAGGGAGGCAAGCCCGACGTCGTCCAGATTGTCCGCAACTA

CAACGCCTACCTTCGGGCCAGCGACGATCTGCCTAAGATGTTCATCGAGTCCGACCCTGGGTTCT

TTTCCAACGCTATTGTCGAGGGAGCTAAGAAGTTCCCTAACACCGAGTTCGTGAAGGTGAAGGG

CCTCCACTTCAGCCAGGAGGACGCTCCAGATGAAATGGGTAAGTACATCAAGAGCTTCGTGGAG

CGCGTGCTGAAGAACGAGCAGTAAGCACACTCTGTCTCCAAGCCGGGAGGGGTGAGTATTGCA

CAGGATTCTGTGGAGGGATAGAGGATACCTTCTAAGGCTTCTTTGTAGACACTGAGTACCCAACT

GAGAGAAGAAATAAAAGATGGTTCTTAGCACAGAAGAAAACACAGGCGTTGATTCAGTCAACT

TACCCAGTGGAGAGACTGGCCTAGGAGAGAAAGACCAGGAGTCTGTGAACAATCTCTGCAGTC

AGTATGAGGAGAAGGTGCGGCCCTGCATTGACCTCATCGACTCCCTGCGGGCTCTGGGTGTGGA

GCAGGACCTGGCCCTGCCTGCCATCGCTGTCATTGGGGACCAGAGTTCTGGGAAGAGCTCTGTA

CTAGAAGCACTGTCTGGAGTGGCCCTCCCCAGAGGCAGTGGTATTGTCACCAGATGCCCTCTGG

TGCTGAAATTGAGGAAGCTGAATGAGGGAGAGGAGTGGAGAGGCAAAGTCTCCTATGATGACA

TCGAAGTGGAGCTCTCTGATCCTTCAGAGGTGGAAGAGGCCATCAACAAGGGTCAGAACTTCAT

TGCTGGGGTAGGTCTGGGGATCAGTGATAAGCTCATTAGCCTGGATGTCAGCTCCCCAAATGTCC

CAGACCTGACTCTCATTGACCTGCCTGGAATTACCAGGGTGGCTGTAGGCAACCAGCCTGCAGA

CATAGGACGCCAGATCAAGAGACTCATCAAGACATACATCCAAAAACAGGAGACCATCAACCT

GGTGGTGGTCCCCAGCAATGTGGACATTGCCACCACAGAGGCTCTGAGCATGGCTCAGGAGGTG

GACCCTGAAGGGGATAGGACCATAGGGATCTTGACCAAGCCCGATCTGGTGGACAGAGGTACT

GAAGACAAGGTCGTGGATGTGGTGCGGAACCTGGTGTACCATCTGAAGAAGGGCTACATGATAG

TCAAGTGCAGAGGTCAGCAGGACATCCAAGAGCAGCTGAGCCTGACTGAGGCTCTTCAGAATG

AGCAAATCTTCTTCAAGGAACACCCTCATTTCAGAGTTCTTCTGGAGGATGGGAAGGCCACAGT

GCCCTGCCTGGCAGAGAGACTGACCGCAGAGCTCATCTCACACATCTGTAAATCTCTTCCTCTGC

TAGAAAATCAAATAAAGGAAAGTCACCAGAGTGCAAGTGAGGAGCTGCAGAAGTACGGTATGG

ACATACCAGAAGATGACAGTGAAAAAACTTTCTTCCTGATCGAAAAAATCAATGCTTTTAACCA

GGACATCACTGCCCTAGTACAAGGGGAAGAGAATGTGGCAGAGGGAGAATGTCGCCTATTCAC

CAGGCTCCGAAAAGAGTTCTTGTCCTGGAGTAAGGAGATCGAAAAGAATTTCGCAAAAAGGTT

ATGCTGTTTTATATAATGAAGTCTGGGCATTTGAAAAGCAGTATCGTGGACGAGAATTGCCAGGG

TTTGTGAATTACAAAACATTTGAGAACATTATAAGAAGACAAATCAAAACCCTGGAAGAGCCAG

CTATAGAAATGCTGCACACGGTCACTGAAATTGTACGAGCTGCCTTTACAAGTGTTTCTGAGAAA

AATTTTTCTGAGTTTTATAACCTCCACAGAACTACCAAGTCCAAACTTGAAGACATCAGATTAGA

ACAAGAAAAGGAAGCAGAGATGTCGATCCGACTCCACTTCAAGATGGAACAAATTATCTACTG

CCAGGACCAGATTTACAGAGGAGCCTTGCAGAAGGTCAGAGAGGAGGAAGCTGAGGAGGAGA

AGAAGACAAAGCATGGCACTTCCAGTTCCTCTCAGTCCCAAGATTTACAGACCTCATCCATGGC

TGAGATCTTCCAGCATCTGAATGCCTACCGCCAGGAGGCTCACAACCGCATCTCCAGCCACGTT

CCCTTGATCATCCAGTATTTCATCCTGAAAATGTTTGCTGAGCGGCTGCAGAAGGGCATGCTCCA

GCTCCTGCAGGACAAGGATTCCTGCAGCTGGCTCCTGAAGGAGCAGAGTGACACAAGCGAGAA

GAGGAAGTTCCTGAAGGAGAGGCTGGCAAGGCTGGCCCAGGCTCGGCGCCGGCTAGCCAAATT

CCCTGGTTAAGCTGGCTCTGTCCTTCCCTGTGTTTCCTGGATTGTGATTCAGGGACAGAAGGGCT

CCTGCCTTTCCTGGTAGCTATACCACCAGCCTTTATGCTCTGATAAGTGCTAGCACTCAGATTTGA

AGGAGCTTTCTGCTCACTCTGAGATGAGGAAGAGAAAGAGGCTCTCAAAGCTCAGCAAGTAGA

TGGGTAGAGGAAGCCACTTTGCTAATAAGACAACGGCTTCACCCTGAGTACTCTTCTTGCCCACC

ATTTAGCACACTGCTAGAAATAAATATTTGATGTACAAATGGGAGCTGCCTGTTGCATTCCTTAAT

AAACCACTTTTCTTTGAAAAAAAAAGCTTGGCATTCCGGTACTGTTGGTAAAGCCACCATGGCC

GATGCTAAGAACATTAAGAAGGGCCCTGCTCCCTTCTACCCTCTGGAGGATGGCACCGCTGGCG

AGCAGCTGCACAAGGCCATGAAGAGGTATGCCCTGGTGCCTGGCACCATTGCCTTCACCGATGC

CCACATTGAGGTGGACATCACCTATGCCGAGTACTTCGAGATGTCTGTGCGCCTGGCCGAGGCC

ATGAAGAGGTACGGCCTGAACACCAACCACCGCATCGTGGTGTGCTCTGAGAACTCTCTGCAGT

TCTTCATGCCAGTGCTGGGCGCCCTGTTCATCGGAGTGGCCGTGGCCCCTGCTAACGACATTTAC

AACGAGCGCGAGCTGCTGAACAGCATGGGCATTTCTCAGCCTACCGTGGTGTTCGTGTCTAAGA

AGGGCCTGCAGAAGATCCTGAACGTGCAGAAGAAGCTGCCTATCATCCAGAAGATCATCATCAT

GGACTCTAAGACCGACTACCAGGGCTTCCAGAGCATGTACACATTCGTGACATCTCATCTGCCTC

CTGGCTTCAACGAGTACGACTTCGTGCCAGAGTCTTTCGACAGGGACAAAACCATTGCCCTGAT

CATGAACAGCTCTGGGTCTACCGGCCTGCCTAAGGGCGTGGCCCTGCCTCATCGCACCGCCTGT

GTGCGCTTCTCTCACGCCCGCGACCCTATTTTCGGCAACCAGATCATCCCCGACACCGCTATTCT

GAGCGTGGTGCCATTCCACCACGGCTTCGGCATGTTCACCACCCTGGGCTACCTGATTTGCGGCT

TTCGGGTGGTGCTGATGTACCGCTTCGAGGAGGAGCTGTTCCTGCGCAGCCTGCAAGACTACAA

AATTCAGTCTGCCCTGCTGGTGCCAACCCTGTTCAGCTTCTTCGCTAAGAGCACCCTGATCGACA

AGTACGACCTGTCTAACCTGCACGAGATTGCCTCTGGCGGCGCCCCACTGTCTAAGGAGGTGGG

CGAAGCCGTGGCCAAGCGCTTTCATCTGCCAGGCATCCGCCAGGGCTACGGCCTGACCGAGAC

AACCAGCGCCATTCTGATTACCCCAGAGGGCGACGACAAGCCTGGCGCCGTGGGCAAGGTGGT

GCCATTCTTCGAGGCCAAGGTGGTGGACCTGGACACCGGCAAGACCCTGGGAGTGAACCAGCG

CGGCGAGCTGTGTGTGCGCGGCCCTATGATTATGTCCGGCTACGTGAATAACCCTGAGGCCACA

AACGCCCTGATCGACAAGGACGGCTGGCTGCACTCTGGCGACATTGCCTACTGGGACGAGGAC

GAGCACTTCTTCATCGTGGACCGCCTGAAGTCTCTGATCAAGTACAAGGGCTACCAGGTGGCCC

CAGCCGAGCTGGAGTCTATCCTGCTGCAGCACCCTAACATTTTCGACGCCGGAGTGGCCGGCCT

GCCCGACGACGATGCCGGCGAGCTGCCTGCCGCCGTCGTCGTGCTGGAACACGGCAAGACCAT

GACCGAGAAGGAGATCGTGGACTATGTGGCCAGCCAGGTGACAACCGCCAAGAAGCTGCGCG

GCGGAGTGGTGTTCGTGGACGAGGTGCCCAAGGGCCTGACCGGCAAGCTGGACGCCCGCAAGA

TCCGCGAGATCCTGATCAAGGCTAAGAAAGGCGGCAAGATCGCCGTGTAATAATTCTAGAGTCG

GGGCGGCCGGCCGCTTCGAGCAGACATGATAAGATACATTGATGAGTTTGGACAAACCACAACT

AGAATGCAGTGAAAAAAATGCTTTATTTGTGAAATTTGTGATGCTATTGCTTTATTTGTAACCATT

ATAAGCTGCAATAAACAAGTTAACAACAACAATTGCATTCATTTTATGTTTCAGGTTCAGGGGGA

GGTGTGGGAGGTTTTTTAAAGCAAGTAAAACCTCTACAAATGTGGTAAAATCGATAAGGATCCA

GGTGGCACTTTTCGGGGAAATGTGCGCGGAACCCCTATTTGTTTATTTTTCTAAATACATTCAAAT

ATGTATCCGCTCATGAGACAATAACCCTGATAAATGCTTCAATAATATTGAAAAAGGAAGAGTAT

GAGTATTCAACATTTCCGTGTCGCCCTTATTCCCTTTTTTGCGGCATTTTGCCTTCCTGTTTTTGCTC

ACCCAGAAACGCTGGTGAAAGTAAAAGATGCTGAAGATCAGTTGGGTGCACGAGTGGGTTACA

TCGAACTGGATCTCAACAGCGGTAAGATCCTTGAGAGTTTTCGCCCCGAAGAACGTTTTCCAAT

GATGAGCACTTTTAAAGTTCTGCTATGTGGCGCGGTATTATCCCGTATTGACGCCGGGCAAGAGC

AACTCGGTCGCCGCATACACTATTCTCAGAATGACTTGGTTGAGTACTCACCAGTCACAGAAAA

GCATCTTACGGATGGCATGACAGTAAGAGAATTATGCAGTGCTGCCATAACCATGAGTGATAACA

CTGCGGCCAACTTACTTCTGACAACGATCGGAGGACCGAAGGAGCTAACCGCTTTTTTGCACAA

CATGGGGGATCATGTAACTCGCCTTGATCGTTGGGAACCGGAGCTGAATGAAGCCATACCAAAC

GACGAGCGTGACACCACGATGCCTGTAGCAATGGCAACAACGTTGCGCAAACTATTAACTGGCG

AACTACTTACTCTAGCTTCCCGGCAACAATTAATAGACTGGATGGAGGCGGATAAAGTTGCAGG

ACCACTTCTGCGCTCGGCCCTTCCGGCTGGCTGGTTTATTGCTGATAAATCTGGAGCCGGTGAGC

GTGGGTCTCGCGGTATCATTGCAGCACTGGGGCCAGATGGTAAGCCCTCCCGTATCGTAGTTATC

TACACGACGGGGAGTCAGGCAACTATGGATGAACGAAATAGACAGATCGCTGAGATAGGTGCC

TCACTGATTAAGCATTGGTAACTGTCAGACCAAGTTTACTCATATATACTTTAGATTGATTTAAAA

CTTCATTTTTAATTTAAAAGGATCTAGGTGAAGATCCTTTTTGATAATCTCATGACCAAAATCCCTT

AACGTGAGTTTTCGTTCCACTGAGCGTCAGACCCCGTAGAAAAGATCAAAGGATCTTCTTGAGA

TCCTTTTTTTCTGCGCGTAATCTGCTGCTTGCAAACAAAAAAACCACCGCTACCAGCGGTGGTTT

GTTTGCCGGATCAAGAGCTACCAACTCTTTTTCCGAAGGTAACTGGCTTCAGCAGAGCGCAGAT

ACCAAATACTGTTCTTCTAGTGTAGCCGTAGTTAGGCCACCACTTCAAGAACTCTGTAGCACCGC

CTACATACCTCGCTCTGCTAATCCTGTTACCAGTGGCTGCTGCCAGTGGCGATAAGTCGTGTCTTA

CCGGGTTGGACTCAAGACGATAGTTACCGGATAAGGCGCAGCGGTCGGGCTGAACGGGGGGTT

CGTGCACACAGCCCAGCTTGGAGCGAACGACCTACACCGAACTGAGATACCTACAGCGTGAGC

TATGAGAAAGCGCCACGCTTCCCGAAGGGAGAAAGGCGGACAGGTATCCGGTAAGCGGCAGG

GTCGGAACAGGAGAGCGCACGAGGGAGCTTCCAGGGGGAAACGCCTGGTATCTTTATAGTCCT

GTCGGGTTTCGCCACCTCTGACTTGAGCGTCGATTTTTGTGATGCTCGTCAGGGGGGCGGAGCCT

ATGGAAAAACGCCAGCAACGCGGCCTTTTTACGGTTCCTGGCCTTTTGCTGGCCTTTTGCTCACA

TGGCTCGAC

2. LncRNA NR_003508 MUT-hRluc-Fluc

AGATCTGCGCAGCACCATGGCCTGAAATAACCTCTGAAAGAGGAACTTGGTTAGGTACCTTCTG

AGGCGGAAAGAACCAGCTGTGGAATGTGTGTCAGTTAGGGTGTGGAAAGTCCCCAGGCTCCCC

AGCAGGCAGAAGTATGCAAAGCATGCATCTCAATTAGTCAGCAACCAGGTGTGGAAAGTCCCC

AGGCTCCCCAGCAGGCAGAAGTATGCAAAGCATGCATCTCAATTAGTCAGCAACCATAGTCCCG

CCCCTAACTCCGCCCATCCCGCCCCTAACTCCGCCCAGTTCCGCCCATTCTCCGCCCCATGGCTG

ACTAATTTTTTTTATTTATGCAGAGGCCGAGGCCGCCTCGGCCTCTGAGCTATTCCAGAAGTAGTG

AGGAGGCTTTTTTGGAGGCCTAGGCTTTTGCAAAAAGCTTGATTCTTCTGACACAACAGTCTCGA

ACTTAAGCTGCAGAAGTTGGTCGTGAGGCACTGGGCAGGTAAGTATCAAGGTTACAAGACAGG

TTTAAGGAGACCAATAGAAACTGGGCTTGTCGAGACAGAGAAGACTCTTGCGTTTCTGATAGGC

ACCTATTGGTCTTACTGACATCCACTTTGCCTTTCTCTCCACAGGTGTCCACTCCCAGTTCAATTA

CAGCTCTTAAGGCTAGAGTACTTAATACGACTCACTATAGGCTAGCCACCATGGCTTCCAAGGTG

TACGACCCCGAGCAACGCAAACGCATGATCACTGGGCCTCAGTGGTGGGCTCGCTGCAAGCAA

ATGAACGTGCTGGACTCCTTCATCAACTACTATGATTCCGAGAAGCACGCCGAGAACGCCGTGA

TTTTTCTGCATGGTAACGCTGCCTCCAGCTACCTGTGGAGGCACGTCGTGCCTCACATCGAGCCC

GTGGCTAGATGCATCATCCCTGATCTGATCGGAATGGGTAAGTCCGGCAAGAGCGGGAATGGCT

CATATCGCCTCCTGGATCACTACAAGTACCTCACCGCTTGGTTCGAGCTGCTGAACCTTCCAAAG

AAAATCATCTTTGTGGGCCACGACTGGGGGGCTTGTCTGGCCTTTCACTACTCCTACGAGCACCA

AGACAAGATCAAGGCCATCGTCCATGCTGAGAGTGTCGTGGACGTGATCGAGTCCTGGGACGA

GTGGCCTGACATCGAGGAGGATATCGCCCTGATCAAGAGCGAAGAGGGCGAGAAAATGGTGCT

TGAGAATAACTTCTTCGTCGAGACCATGCTCCCAAGCAAGATCATGCGGAAACTGGAGCCTGAG

GAGTTCGCTGCCTACCTGGAGCCATTCAAGGAGAAGGGCGAGGTTAGACGGCCTACCCTCTCCT

GGCCTCGCGAGATCCCTCTCGTTAAGGGAGGCAAGCCCGACGTCGTCCAGATTGTCCGCAACTA

CAACGCCTACCTTCGGGCCAGCGACGATCTGCCTAAGATGTTCATCGAGTCCGACCCTGGGTTCT

TTTCCAACGCTATTGTCGAGGGAGCTAAGAAGTTCCCTAACACCGAGTTCGTGAAGGTGAAGGG

CCTCCACTTCAGCCAGGAGGACGCTCCAGATGAAATGGGTAAGTACATCAAGAGCTTCGTGGAG

CGCGTGCTGAAGAACGAGCAGTAAGCACACTCTGTCTCCAAGCCGGGAGGGGTGAGTATTGCA

CAGGATTCTGTGGAGGGATAGAGGATACCTTCTAAGGCTTCTTTGTAGACACTGAGTACCCAACT

GAGAGAAGAAATAAAAGATGGTTCTTAGCACAGAAGAAAACACAGGCGTTGATTCAGTCAACT

TACCCAGTGGAGAGACTGGCCTAGGAGAGAAAGACCAGGAGTCTGTGAACAATCTCTGCAGTC

AGTATGAGGAGAAGGTGCGGCCCTGCATTGACCTCATCGACTCCCTGCGGGCTCTGGGTGTGGA

GCAGGACCTGGCCCTGCCTGCCATCGCTGTCATTGGGGACCAGAGTTCTGGGAAGAGCTCTGTA

CTAGAAGCACTGTCTGGAGTGGCCCTCCCCAGAGGCAGTGGTATTGTCACCAGATGCCCTCTGG

TGCTGAAATTGAGGAAGCTGAATGAGGGAGAGGAGTGGAGAGGCAAAGTCTCCTATGATGACA

TCGAAGTGGAGCTCTCTGATCCTTCAGAGGTGGAAGAGGCCATCAACAAGGGTCAGAACTTCAT

TGCTGGGGTAGGTCTGGGGATCAGTGATAAGCTCATTAGCCTGGATGTCAGCTCCCCAAATGTCC

CAGACCTGACTCTCATTGACCTGCCTGGAATTACCAGGGTGGCTGTAGGCAACCAGCCTGCAGA

CATAGGACGCCAGATCAAGAGACTCATCAAGACATACATCCAAAAACAGGAGACCATCAACCT

GGTGGTGGTCCCCAGCAATGTGGACATTGCCACCACAGAGGCTCTGAGCATGGCTCAGGAGGTG

GACCCTGAAGGGGATAGGACCATAGGGATCTTGACCAAGCCCGATCTGGTGGACAGAGGTACT

GAAGACAAGGTCGTGGATGTGGTGCGGAACCTGGTGTACCATCTGAAGAAGGGCTACATGATAG

TCAAGTGCAGAGGTCAGCAGGACATCCAAGAGGGTCGACTCGGACTGACAGGCTCTTCAGAATG

AGCAAATCTTCTTCAAGGAACACCCTCATTTCAGAGTTCTTCTGGAGGATGGGAAGGCCACAGT

GCCCTGCCTGGCAGAGAGACTGACCGCAGAGCTCATCTCACACATCTGTAAATCTCTTCCTCTGC

TAGAAAATCAAATAAAGGAAAGTCACCAGAGTGCAAGTGAGGAGCTGCAGAAGTACGGTATGG

ACATACCAGAAGATGACAGTGAAAAAACTTTCTTCCTGATCGAAAAAATCAATGCTTTTAACCA

GGACATCACTGCCCTAGTACAAGGGGAAGAGAATGTGGCAGAGGGAGAATGTCGCCTATTCAC

CAGGCTCCGAAAAGAGTTCTTGTCCTGGAGTAAGGAGATCGAAAAGAATTTCGCAAAAAGGTT

ATGCTGTTTTATATAATGAAGTCTGGGCATTTGAAAAGCAGTATCGTGGACGAGAATTGCCAGGG

TTTGTGAATTACAAAACATTTGAGAACATTATAAGAAGACAAATCAAAACCCTGGAAGAGCCAG

CTATAGAAATGCTGCACACGGTCACTGAAATTGTACGAGCTGCCTTTACAAGTGTTTCTGAGAAA

AATTTTTCTGAGTTTTATAACCTCCACAGAACTACCAAGTCCAAACTTGAAGACATCAGATTAGA

ACAAGAAAAGGAAGCAGAGATGTCGATCCGACTCCACTTCAAGATGGAACAAATTATCTACTG

CCAGGACCAGATTTACAGAGGAGCCTTGCAGAAGGTCAGAGAGGAGGAAGCTGAGGAGGAGA

AGAAGACAAAGCATGGCACTTCCAGTTCCTCTCAGTCCCAAGATTTACAGACCTCATCCATGGC

TGAGATCTTCCAGCATCTGAATGCCTACCGCCAGGAGGCTCACAACCGCATCTCCAGCCACGTT

CCCTTGATCATCCAGTATTTCATCCTGAAAATGTTTGCTGAGCGGCTGCAGAAGGGCATGCTCCA

GCTCCTGCAGGACAAGGATTCCTGCAGCTGGCTCCTGAAGGAGCAGAGTGACACAAGCGAGAA

GAGGAAGTTCCTGAAGGAGAGGCTGGCAAGGCTGGCCCAGGCTCGGCGCCGGCTAGCCAAATT

CCCTGGTTAAGCTGGCTCTGTCCTTCCCTGTGTTTCCTGGATTGTGATTCAGGGACAGAAGGGCT

CCTGCCTTTCCTGGTAGCTATACCACCAGCCTTTATGCTCTGATAAGTGCTAGCACTCAGATTTGA

AGGAGCTTTCTGCTCACTCTGAGATGAGGAAGAGAAAGAGGCTCTCAAAGCTCAGCAAGTAGA

TGGGTAGAGGAAGCCACTTTGCTAATAAGACAACGGCTTCACCCTGAGTACTCTTCTTGCCCACC

ATTTAGCACACTGCTAGAAATAAATATTTGATGTACAAATGGGAGCTGCCTGTTGCATTCCTTAAT

AAACCACTTTTCTTTGAAAAAAAAAGCTTGGCATTCCGGTACTGTTGGTAAAGCCACCATGGCC

GATGCTAAGAACATTAAGAAGGGCCCTGCTCCCTTCTACCCTCTGGAGGATGGCACCGCTGGCG

AGCAGCTGCACAAGGCCATGAAGAGGTATGCCCTGGTGCCTGGCACCATTGCCTTCACCGATGC

CCACATTGAGGTGGACATCACCTATGCCGAGTACTTCGAGATGTCTGTGCGCCTGGCCGAGGCC

ATGAAGAGGTACGGCCTGAACACCAACCACCGCATCGTGGTGTGCTCTGAGAACTCTCTGCAGT

TCTTCATGCCAGTGCTGGGCGCCCTGTTCATCGGAGTGGCCGTGGCCCCTGCTAACGACATTTAC

AACGAGCGCGAGCTGCTGAACAGCATGGGCATTTCTCAGCCTACCGTGGTGTTCGTGTCTAAGA

AGGGCCTGCAGAAGATCCTGAACGTGCAGAAGAAGCTGCCTATCATCCAGAAGATCATCATCAT

GGACTCTAAGACCGACTACCAGGGCTTCCAGAGCATGTACACATTCGTGACATCTCATCTGCCTC

CTGGCTTCAACGAGTACGACTTCGTGCCAGAGTCTTTCGACAGGGACAAAACCATTGCCCTGAT

CATGAACAGCTCTGGGTCTACCGGCCTGCCTAAGGGCGTGGCCCTGCCTCATCGCACCGCCTGT

GTGCGCTTCTCTCACGCCCGCGACCCTATTTTCGGCAACCAGATCATCCCCGACACCGCTATTCT

GAGCGTGGTGCCATTCCACCACGGCTTCGGCATGTTCACCACCCTGGGCTACCTGATTTGCGGCT

TTCGGGTGGTGCTGATGTACCGCTTCGAGGAGGAGCTGTTCCTGCGCAGCCTGCAAGACTACAA

AATTCAGTCTGCCCTGCTGGTGCCAACCCTGTTCAGCTTCTTCGCTAAGAGCACCCTGATCGACA

AGTACGACCTGTCTAACCTGCACGAGATTGCCTCTGGCGGCGCCCCACTGTCTAAGGAGGTGGG

CGAAGCCGTGGCCAAGCGCTTTCATCTGCCAGGCATCCGCCAGGGCTACGGCCTGACCGAGAC

AACCAGCGCCATTCTGATTACCCCAGAGGGCGACGACAAGCCTGGCGCCGTGGGCAAGGTGGT

GCCATTCTTCGAGGCCAAGGTGGTGGACCTGGACACCGGCAAGACCCTGGGAGTGAACCAGCG

CGGCGAGCTGTGTGTGCGCGGCCCTATGATTATGTCCGGCTACGTGAATAACCCTGAGGCCACA

AACGCCCTGATCGACAAGGACGGCTGGCTGCACTCTGGCGACATTGCCTACTGGGACGAGGAC

GAGCACTTCTTCATCGTGGACCGCCTGAAGTCTCTGATCAAGTACAAGGGCTACCAGGTGGCCC

CAGCCGAGCTGGAGTCTATCCTGCTGCAGCACCCTAACATTTTCGACGCCGGAGTGGCCGGCCT

GCCCGACGACGATGCCGGCGAGCTGCCTGCCGCCGTCGTCGTGCTGGAACACGGCAAGACCAT

GACCGAGAAGGAGATCGTGGACTATGTGGCCAGCCAGGTGACAACCGCCAAGAAGCTGCGCG

GCGGAGTGGTGTTCGTGGACGAGGTGCCCAAGGGCCTGACCGGCAAGCTGGACGCCCGCAAGA

TCCGCGAGATCCTGATCAAGGCTAAGAAAGGCGGCAAGATCGCCGTGTAATAATTCTAGAGTCG

GGGCGGCCGGCCGCTTCGAGCAGACATGATAAGATACATTGATGAGTTTGGACAAACCACAACT

AGAATGCAGTGAAAAAAATGCTTTATTTGTGAAATTTGTGATGCTATTGCTTTATTTGTAACCATT

ATAAGCTGCAATAAACAAGTTAACAACAACAATTGCATTCATTTTATGTTTCAGGTTCAGGGGGA

GGTGTGGGAGGTTTTTTAAAGCAAGTAAAACCTCTACAAATGTGGTAAAATCGATAAGGATCCA

GGTGGCACTTTTCGGGGAAATGTGCGCGGAACCCCTATTTGTTTATTTTTCTAAATACATTCAAAT

ATGTATCCGCTCATGAGACAATAACCCTGATAAATGCTTCAATAATATTGAAAAAGGAAGAGTAT

GAGTATTCAACATTTCCGTGTCGCCCTTATTCCCTTTTTTGCGGCATTTTGCCTTCCTGTTTTTGCTC

ACCCAGAAACGCTGGTGAAAGTAAAAGATGCTGAAGATCAGTTGGGTGCACGAGTGGGTTACA

TCGAACTGGATCTCAACAGCGGTAAGATCCTTGAGAGTTTTCGCCCCGAAGAACGTTTTCCAAT

GATGAGCACTTTTAAAGTTCTGCTATGTGGCGCGGTATTATCCCGTATTGACGCCGGGCAAGAGC

AACTCGGTCGCCGCATACACTATTCTCAGAATGACTTGGTTGAGTACTCACCAGTCACAGAAAA

GCATCTTACGGATGGCATGACAGTAAGAGAATTATGCAGTGCTGCCATAACCATGAGTGATAACA

CTGCGGCCAACTTACTTCTGACAACGATCGGAGGACCGAAGGAGCTAACCGCTTTTTTGCACAA

CATGGGGGATCATGTAACTCGCCTTGATCGTTGGGAACCGGAGCTGAATGAAGCCATACCAAAC

GACGAGCGTGACACCACGATGCCTGTAGCAATGGCAACAACGTTGCGCAAACTATTAACTGGCG

AACTACTTACTCTAGCTTCCCGGCAACAATTAATAGACTGGATGGAGGCGGATAAAGTTGCAGG

ACCACTTCTGCGCTCGGCCCTTCCGGCTGGCTGGTTTATTGCTGATAAATCTGGAGCCGGTGAGC

GTGGGTCTCGCGGTATCATTGCAGCACTGGGGCCAGATGGTAAGCCCTCCCGTATCGTAGTTATC

TACACGACGGGGAGTCAGGCAACTATGGATGAACGAAATAGACAGATCGCTGAGATAGGTGCC

TCACTGATTAAGCATTGGTAACTGTCAGACCAAGTTTACTCATATATACTTTAGATTGATTTAAAA

CTTCATTTTTAATTTAAAAGGATCTAGGTGAAGATCCTTTTTGATAATCTCATGACCAAAATCCCTT

AACGTGAGTTTTCGTTCCACTGAGCGTCAGACCCCGTAGAAAAGATCAAAGGATCTTCTTGAGA

TCCTTTTTTTCTGCGCGTAATCTGCTGCTTGCAAACAAAAAAACCACCGCTACCAGCGGTGGTTT

GTTTGCCGGATCAAGAGCTACCAACTCTTTTTCCGAAGGTAACTGGCTTCAGCAGAGCGCAGAT

ACCAAATACTGTTCTTCTAGTGTAGCCGTAGTTAGGCCACCACTTCAAGAACTCTGTAGCACCGC

CTACATACCTCGCTCTGCTAATCCTGTTACCAGTGGCTGCTGCCAGTGGCGATAAGTCGTGTCTTA

CCGGGTTGGACTCAAGACGATAGTTACCGGATAAGGCGCAGCGGTCGGGCTGAACGGGGGGTT

CGTGCACACAGCCCAGCTTGGAGCGAACGACCTACACCGAACTGAGATACCTACAGCGTGAGC

TATGAGAAAGCGCCACGCTTCCCGAAGGGAGAAAGGCGGACAGGTATCCGGTAAGCGGCAGG

GTCGGAACAGGAGAGCGCACGAGGGAGCTTCCAGGGGGAAACGCCTGGTATCTTTATAGTCCT

GTCGGGTTTCGCCACCTCTGACTTGAGCGTCGATTTTTGTGATGCTCGTCAGGGGGGCGGAGCCT

ATGGAAAAACGCCAGCAACGCGGCCTTTTTACGGTTCCTGGCCTTTTGCTGGCCTTTTGCTCACA

TGGCTCGAC

3. LC3 WT-hRluc-Fluc

AGATCTGCGCAGCACCATGGCCTGAAATAACCTCTGAAAGAGGAACTTGGTTAGGTACCTTCTG

AGGCGGAAAGAACCAGCTGTGGAATGTGTGTCAGTTAGGGTGTGGAAAGTCCCCAGGCTCCCC

AGCAGGCAGAAGTATGCAAAGCATGCATCTCAATTAGTCAGCAACCAGGTGTGGAAAGTCCCC

AGGCTCCCCAGCAGGCAGAAGTATGCAAAGCATGCATCTCAATTAGTCAGCAACCATAGTCCC

GCCCCTAACTCCGCCCATCCCGCCCCTAACTCCGCCCAGTTCCGCCCATTCTCCGCCCCATGGCT

GACTAATTTTTTTTATTTATGCAGAGGCCGAGGCCGCCTCGGCCTCTGAGCTATTCCAGAAGTAG

TGAGGAGGCTTTTTTGGAGGCCTAGGCTTTTGCAAAAAGCTTGATTCTTCTGACACAACAGTCTC

GAACTTAAGCTGCAGAAGTTGGTCGTGAGGCACTGGGCAGGTAAGTATCAAGGTTACAAGACA

GGTTTAAGGAGACCAATAGAAACTGGGCTTGTCGAGACAGAGAAGACTCTTGCGTTTCTGATA

GGCACCTATTGGTCTTACTGACATCCACTTTGCCTTTCTCTCCACAGGTGTCCACTCCCAGTTCAA

TTACAGCTCTTAAGGCTAGAGTACTTAATACGACTCACTATAGGCTAGCCACCATGGCTTCCAA

GGTGTACGACCCCGAGCAACGCAAACGCATGATCACTGGGCCTCAGTGGTGGGCTCGCTGCAA

GCAAATGAACGTGCTGGACTCCTTCATCAACTACTATGATTCCGAGAAGCACGCCGAGAACGC

CGTGATTTTTCTGCATGGTAACGCTGCCTCCAGCTACCTGTGGAGGCACGTCGTGCCTCACATCG

AGCCCGTGGCTAGATGCATCATCCCTGATCTGATCGGAATGGGTAAGTCCGGCAAGAGCGGGA

ATGGCTCATATCGCCTCCTGGATCACTACAAGTACCTCACCGCTTGGTTCGAGCTGCTGAACCTT

CCAAAGAAAATCATCTTTGTGGGCCACGACTGGGGGGCTTGTCTGGCCTTTCACTACTCCTACG

AGCACCAAGACAAGATCAAGGCCATCGTCCATGCTGAGAGTGTCGTGGACGTGATCGAGTCCT

GGGACGAGTGGCCTGACATCGAGGAGGATATCGCCCTGATCAAGAGCGAAGAGGGCGAGAAA

ATGGTGCTTGAGAATAACTTCTTCGTCGAGACCATGCTCCCAAGCAAGATCATGCGGAAACTGG

AGCCTGAGGAGTTCGCTGCCTACCTGGAGCCATTCAAGGAGAAGGGCGAGGTTAGACGGCCTA

CCCTCTCCTGGCCTCGCGAGATCCCTCTCGTTAAGGGAGGCAAGCCCGACGTCGTCCAGATTGT

CCGCAACTACAACGCCTACCTTCGGGCCAGCGACGATCTGCCTAAGATGTTCATCGAGTCCGAC

CCTGGGTTCTTTTCCAACGCTATTGTCGAGGGAGCTAAGAAGTTCCCTAACACCGAGTTCGTGA

AGGTGAAGGGCCTCCACTTCAGCCAGGAGGACGCTCCAGATGAAATGGGTAAGTACATCAAGA

GCTTCGTGGAGCGCGTGCTGAAGAACGAGCAGTAAGTCAAGAGGAGGGGAGGGGGGTGGCTG

GGAGTTCTGGTCAGGTTCTCCCCAGGGAGGTCCTGGCTCCTAAACTAAGCTATTTCAGTCCCCA

GTGGATTAGGCAGAGATGTGACACCCACTCCCCCCCCCAGGTAGGGGCCACCAGCCAGCCTAC

CACATCCTGGGTAGGTCCTGGGCCAGTCATGTTCGGGTTGCTCTTTTGGGTGCTGGCTGGGTTGG

GAGTGGGTGGGGAGCAGCATCCCTGCTCTGTGGGGTTTGTCATTTTGTTAGGCCCTTGCCTGTCT

GCCCATCTTGCCCCTCATCCACCTGAGGCTTTGCCTCCTGCCAGGACCTGCCCCACCCCTGAAA

GGCTGGCTCCCCTTGTCCTGACTCGGTGTATGGATCTGTGGTCATTTCCTCTGCAGAAAGAATAA

AGACTGCTCAGGCCTGCCTGGCCAAAAAAAAAAAAAAAAAAAAGCTTGGCATTCCGGTACTGT

TGGTAAAGCCACCATGGCCGATGCTAAGAACATTAAGAAGGGCCCTGCTCCCTTCTACCCTCTG

GAGGATGGCACCGCTGGCGAGCAGCTGCACAAGGCCATGAAGAGGTATGCCCTGGTGCCTGGC

ACCATTGCCTTCACCGATGCCCACATTGAGGTGGACATCACCTATGCCGAGTACTTCGAGATGT

CTGTGCGCCTGGCCGAGGCCATGAAGAGGTACGGCCTGAACACCAACCACCGCATCGTGGTGT

GCTCTGAGAACTCTCTGCAGTTCTTCATGCCAGTGCTGGGCGCCCTGTTCATCGGAGTGGCCGTG

GCCCCTGCTAACGACATTTACAACGAGCGCGAGCTGCTGAACAGCATGGGCATTTCTCAGCCTA

CCGTGGTGTTCGTGTCTAAGAAGGGCCTGCAGAAGATCCTGAACGTGCAGAAGAAGCTGCCTA

TCATCCAGAAGATCATCATCATGGACTCTAAGACCGACTACCAGGGCTTCCAGAGCATGTACA

CATTCGTGACATCTCATCTGCCTCCTGGCTTCAACGAGTACGACTTCGTGCCAGAGTCTTTCGAC

AGGGACAAAACCATTGCCCTGATCATGAACAGCTCTGGGTCTACCGGCCTGCCTAAGGGCGTG

GCCCTGCCTCATCGCACCGCCTGTGTGCGCTTCTCTCACGCCCGCGACCCTATTTTCGGCAACCA

GATCATCCCCGACACCGCTATTCTGAGCGTGGTGCCATTCCACCACGGCTTCGGCATGTTCACC

ACCCTGGGCTACCTGATTTGCGGCTTTCGGGTGGTGCTGATGTACCGCTTCGAGGAGGAGCTGTT

CCTGCGCAGCCTGCAAGACTACAAAATTCAGTCTGCCCTGCTGGTGCCAACCCTGTTCAGCTTC

TTCGCTAAGAGCACCCTGATCGACAAGTACGACCTGTCTAACCTGCACGAGATTGCCTCTGGCG

GCGCCCCACTGTCTAAGGAGGTGGGCGAAGCCGTGGCCAAGCGCTTTCATCTGCCAGGCATCC

GCCAGGGCTACGGCCTGACCGAGACAACCAGCGCCATTCTGATTACCCCAGAGGGCGACGACA

AGCCTGGCGCCGTGGGCAAGGTGGTGCCATTCTTCGAGGCCAAGGTGGTGGACCTGGACACCG

GCAAGACCCTGGGAGTGAACCAGCGCGGCGAGCTGTGTGTGCGCGGCCCTATGATTATGTCCG

GCTACGTGAATAACCCTGAGGCCACAAACGCCCTGATCGACAAGGACGGCTGGCTGCACTCTG

GCGACATTGCCTACTGGGACGAGGACGAGCACTTCTTCATCGTGGACCGCCTGAAGTCTCTGAT

CAAGTACAAGGGCTACCAGGTGGCCCCAGCCGAGCTGGAGTCTATCCTGCTGCAGCACCCTAA

CATTTTCGACGCCGGAGTGGCCGGCCTGCCCGACGACGATGCCGGCGAGCTGCCTGCCGCCGTC

GTCGTGCTGGAACACGGCAAGACCATGACCGAGAAGGAGATCGTGGACTATGTGGCCAGCCA

GGTGACAACCGCCAAGAAGCTGCGCGGCGGAGTGGTGTTCGTGGACGAGGTGCCCAAGGGCCT

GACCGGCAAGCTGGACGCCCGCAAGATCCGCGAGATCCTGATCAAGGCTAAGAAAGGCGGCA

AGATCGCCGTGTAATAATTCTAGAGTCGGGGCGGCCGGCCGCTTCGAGCAGACATGATAAGAT

ACATTGATGAGTTTGGACAAACCACAACTAGAATGCAGTGAAAAAAATGCTTTATTTGTGAAAT

TTGTGATGCTATTGCTTTATTTGTAACCATTATAAGCTGCAATAAACAAGTTAACAACAACAATT

GCATTCATTTTATGTTTCAGGTTCAGGGGGAGGTGTGGGAGGTTTTTTAAAGCAAGTAAAACCTC

TACAAATGTGGTAAAATCGATAAGGATCCAGGTGGCACTTTTCGGGGAAATGTGCGCGGAACC

CCTATTTGTTTATTTTTCTAAATACATTCAAATATGTATCCGCTCATGAGACAATAACCCTGATA

AATGCTTCAATAATATTGAAAAAGGAAGAGTATGAGTATTCAACATTTCCGTGTCGCCCTTATT

CCCTTTTTTGCGGCATTTTGCCTTCCTGTTTTTGCTCACCCAGAAACGCTGGTGAAAGTAAAAGA

TGCTGAAGATCAGTTGGGTGCACGAGTGGGTTACATCGAACTGGATCTCAACAGCGGTAAGAT

CCTTGAGAGTTTTCGCCCCGAAGAACGTTTTCCAATGATGAGCACTTTTAAAGTTCTGCTATGTG

GCGCGGTATTATCCCGTATTGACGCCGGGCAAGAGCAACTCGGTCGCCGCATACACTATTCTCA

GAATGACTTGGTTGAGTACTCACCAGTCACAGAAAAGCATCTTACGGATGGCATGACAGTAAG

AGAATTATGCAGTGCTGCCATAACCATGAGTGATAACACTGCGGCCAACTTACTTCTGACAACG

ATCGGAGGACCGAAGGAGCTAACCGCTTTTTTGCACAACATGGGGGATCATGTAACTCGCCTTG

ATCGTTGGGAACCGGAGCTGAATGAAGCCATACCAAACGACGAGCGTGACACCACGATGCCTG

TAGCAATGGCAACAACGTTGCGCAAACTATTAACTGGCGAACTACTTACTCTAGCTTCCCGGCA

ACAATTAATAGACTGGATGGAGGCGGATAAAGTTGCAGGACCACTTCTGCGCTCGGCCCTTCCG

GCTGGCTGGTTTATTGCTGATAAATCTGGAGCCGGTGAGCGTGGGTCTCGCGGTATCATTGCAG

CACTGGGGCCAGATGGTAAGCCCTCCCGTATCGTAGTTATCTACACGACGGGGAGTCAGGCAA

CTATGGATGAACGAAATAGACAGATCGCTGAGATAGGTGCCTCACTGATTAAGCATTGGTAAC

TGTCAGACCAAGTTTACTCATATATACTTTAGATTGATTTAAAACTTCATTTTTAATTTAAAAGG

ATCTAGGTGAAGATCCTTTTTGATAATCTCATGACCAAAATCCCTTAACGTGAGTTTTCGTTCCA

CTGAGCGTCAGACCCCGTAGAAAAGATCAAAGGATCTTCTTGAGATCCTTTTTTTCTGCGCGTA

ATCTGCTGCTTGCAAACAAAAAAACCACCGCTACCAGCGGTGGTTTGTTTGCCGGATCAAGAGC

TACCAACTCTTTTTCCGAAGGTAACTGGCTTCAGCAGAGCGCAGATACCAAATACTGTTCTTCTA

GTGTAGCCGTAGTTAGGCCACCACTTCAAGAACTCTGTAGCACCGCCTACATACCTCGCTCTGC

TAATCCTGTTACCAGTGGCTGCTGCCAGTGGCGATAAGTCGTGTCTTACCGGGTTGGACTCAAG

ACGATAGTTACCGGATAAGGCGCAGCGGTCGGGCTGAACGGGGGGTTCGTGCACACAGCCCAG

CTTGGAGCGAACGACCTACACCGAACTGAGATACCTACAGCGTGAGCTATGAGAAAGCGCCAC

GCTTCCCGAAGGGAGAAAGGCGGACAGGTATCCGGTAAGCGGCAGGGTCGGAACAGGAGAGC

GCACGAGGGAGCTTCCAGGGGGAAACGCCTGGTATCTTTATAGTCCTGTCGGGTTTCGCCACCT

CTGACTTGAGCGTCGATTTTTGTGATGCTCGTCAGGGGGGCGGAGCCTATGGAAAAACGCCAGC

AACGCGGCCTTTTTACGGTTCCTGGCCTTTTGCTGGCCTTTTGCTCACATGGCTCGAC

4. LC3 MUT-hRluc-Fluc

AGATCTGCGCAGCACCATGGCCTGAAATAACCTCTGAAAGAGGAACTTGGTTAGGTACCTTCTG

AGGCGGAAAGAACCAGCTGTGGAATGTGTGTCAGTTAGGGTGTGGAAAGTCCCCAGGCTCCCC

AGCAGGCAGAAGTATGCAAAGCATGCATCTCAATTAGTCAGCAACCAGGTGTGGAAAGTCCCC

AGGCTCCCCAGCAGGCAGAAGTATGCAAAGCATGCATCTCAATTAGTCAGCAACCATAGTCCCG

CCCCTAACTCCGCCCATCCCGCCCCTAACTCCGCCCAGTTCCGCCCATTCTCCGCCCCATGGCTG

ACTAATTTTTTTTATTTATGCAGAGGCCGAGGCCGCCTCGGCCTCTGAGCTATTCCAGAAGTAGTG

AGGAGGCTTTTTTGGAGGCCTAGGCTTTTGCAAAAAGCTTGATTCTTCTGACACAACAGTCTCGA

ACTTAAGCTGCAGAAGTTGGTCGTGAGGCACTGGGCAGGTAAGTATCAAGGTTACAAGACAGG

TTTAAGGAGACCAATAGAAACTGGGCTTGTCGAGACAGAGAAGACTCTTGCGTTTCTGATAGGC

ACCTATTGGTCTTACTGACATCCACTTTGCCTTTCTCTCCACAGGTGTCCACTCCCAGTTCAATTA

CAGCTCTTAAGGCTAGAGTACTTAATACGACTCACTATAGGCTAGCCACCATGGCTTCCAAGGTG

TACGACCCCGAGCAACGCAAACGCATGATCACTGGGCCTCAGTGGTGGGCTCGCTGCAAGCAA

ATGAACGTGCTGGACTCCTTCATCAACTACTATGATTCCGAGAAGCACGCCGAGAACGCCGTGA

TTTTTCTGCATGGTAACGCTGCCTCCAGCTACCTGTGGAGGCACGTCGTGCCTCACATCGAGCCC

GTGGCTAGATGCATCATCCCTGATCTGATCGGAATGGGTAAGTCCGGCAAGAGCGGGAATGGCT

CATATCGCCTCCTGGATCACTACAAGTACCTCACCGCTTGGTTCGAGCTGCTGAACCTTCCAAAG

AAAATCATCTTTGTGGGCCACGACTGGGGGGCTTGTCTGGCCTTTCACTACTCCTACGAGCACCA

AGACAAGATCAAGGCCATCGTCCATGCTGAGAGTGTCGTGGACGTGATCGAGTCCTGGGACGA

GTGGCCTGACATCGAGGAGGATATCGCCCTGATCAAGAGCGAAGAGGGCGAGAAAATGGTGCT

TGAGAATAACTTCTTCGTCGAGACCATGCTCCCAAGCAAGATCATGCGGAAACTGGAGCCTGAG

GAGTTCGCTGCCTACCTGGAGCCATTCAAGGAGAAGGGCGAGGTTAGACGGCCTACCCTCTCCT

GGCCTCGCGAGATCCCTCTCGTTAAGGGAGGCAAGCCCGACGTCGTCCAGATTGTCCGCAACTA

CAACGCCTACCTTCGGGCCAGCGACGATCTGCCTAAGATGTTCATCGAGTCCGACCCTGGGTTCT

TTTCCAACGCTATTGTCGAGGGAGCTAAGAAGTTCCCTAACACCGAGTTCGTGAAGGTGAAGGG

CCTCCACTTCAGCCAGGAGGACGCTCCAGATGAAATGGGTAAGTACATCAAGAGCTTCGTGGAG

CGCGTGCTGAAGAACGAGCAGTAAGTCAAGAGGAGGGGAGGGGGGTGGCTGGGAGTTCTGGT

CAGGTTCAGGGGTCCGAGCTGGACGGAGCTAAACTAAGCTATTTCAGTCCCCAGTGGATTAGG

CAGAGATGTGACACCCACTCCCCCCCCAGGTAGGGGCCACCAGCCAGCCTACCACATCCTGG

GTAGGTCCTGGGCCAGTCATGTTCGGGTTGCTCTTTGGGTGCTGGCTGGGTTGGGAGTGGGTGG

GGAGCAGCATCCCTGCTCTGTGGGGTTTGTCATTTTGTTAGGCCCTTGCCTGTCTGCCCATCTTGC

CCCTCATCCACCTGAGGCTTTGCCTCCTGCCAGGACCTGCCCCACCCCTGAAAGGCTGGCTCCC

CTTGTCCTGACTCGGTGTATGGATCTGTGGTCATTTCCTCTGCAGAAAGAATAAAGACTGCTCAG

GCCTGCCTGGCCAAAAAAAAAAAAAAAAAAAAGCTTGGCATTCCGGTACTGTTGGTAAAGCCA

CCATGGCCGATGCTAAGAACATTAAGAAGGGCCCTGCTCCCTTCTACCCTCTGGAGGATGGCAC

CGCTGGCGAGCAGCTGCACAAGGCCATGAAGAGGTATGCCCTGGTGCCTGGCACCATTGCCTTC

ACCGATGCCCACATTGAGGTGGACATCACCTATGCCGAGTACTTCGAGATGTCTGTGCGCCTGGC

CGAGGCCATGAAGAGGTACGGCCTGAACACCAACCACCGCATCGTGGTGTGCTCTGAGAACTC

TCTGCAGTTCTTCATGCCAGTGCTGGGCGCCCTGTTCATCGGAGTGGCCGTGGCCCCTGCTAACG

ACATTTACAACGAGCGCGAGCTGCTGAACAGCATGGGCATTTCTCAGCCTACCGTGGTGTTCGTG

TCTAAGAAGGGCCTGCAGAAGATCCTGAACGTGCAGAAGAAGCTGCCTATCATCCAGAAGATC

ATCATCATGGACTCTAAGACCGACTACCAGGGCTTCCAGAGCATGTACACATTCGTGACATCTCA

TCTGCCTCCTGGCTTCAACGAGTACGACTTCGTGCCAGAGTCTTTCGACAGGGACAAAACCATT

GCCCTGATCATGAACAGCTCTGGGTCTACCGGCCTGCCTAAGGGCGTGGCCCTGCCTCATCGCA

CCGCCTGTGTGCGCTTCTCTCACGCCCGCGACCCTATTTTCGGCAACCAGATCATCCCCGACACC

GCTATTCTGAGCGTGGTGCCATTCCACCACGGCTTCGGCATGTTCACCACCCTGGGCTACCTGAT

TTGCGGCTTTCGGGTGGTGCTGATGTACCGCTTCGAGGAGGAGCTGTTCCTGCGCAGCCTGCAA

GACTACAAAATTCAGTCTGCCCTGCTGGTGCCAACCCTGTTCAGCTTCTTCGCTAAGAGCACCCT

GATCGACAAGTACGACCTGTCTAACCTGCACGAGATTGCCTCTGGCGGCGCCCCACTGTCTAAG

GAGGTGGGCGAAGCCGTGGCCAAGCGCTTTCATCTGCCAGGCATCCGCCAGGGCTACGGCCTG

ACCGAGACAACCAGCGCCATTCTGATTACCCCAGAGGGCGACGACAAGCCTGGCGCCGTGGGC

AAGGTGGTGCCATTCTTCGAGGCCAAGGTGGTGGACCTGGACACCGGCAAGACCCTGGGAGTG

AACCAGCGCGGCGAGCTGTGTGTGCGCGGCCCTATGATTATGTCCGGCTACGTGAATAACCCTG

AGGCCACAAACGCCCTGATCGACAAGGACGGCTGGCTGCACTCTGGCGACATTGCCTACTGGG

ACGAGGACGAGCACTTCTTCATCGTGGACCGCCTGAAGTCTCTGATCAAGTACAAGGGCTACCA

GGTGGCCCCAGCCGAGCTGGAGTCTATCCTGCTGCAGCACCCTAACATTTTCGACGCCGGAGTG

GCCGGCCTGCCCGACGACGATGCCGGCGAGCTGCCTGCCGCCGTCGTCGTGCTGGAACACGGC

AAGACCATGACCGAGAAGGAGATCGTGGACTATGTGGCCAGCCAGGTGACAACCGCCAAGAA

GCTGCGCGGCGGAGTGGTGTTCGTGGACGAGGTGCCCAAGGGCCTGACCGGCAAGCTGGACGC

CCGCAAGATCCGCGAGATCCTGATCAAGGCTAAGAAAGGCGGCAAGATCGCCGTGTAATAATTC

TAGAGTCGGGGCGGCCGGCCGCTTCGAGCAGACATGATAAGATACATTGATGAGTTTGGACAAA

CCACAACTAGAATGCAGTGAAAAAAATGCTTTATTTGTGAAATTTGTGATGCTATTGCTTTATTTG

TAACCATTATAAGCTGCAATAAACAAGTTAACAACAACAATTGCATTCATTTTATGTTTCAGGTTC

AGGGGGAGGTGTGGGAGGTTTTTTAAAGCAAGTAAAACCTCTACAAATGTGGTAAAATCGATAA

GGATCCAGGTGGCACTTTTCGGGGAAATGTGCGCGGAACCCCTATTTGTTTATTTTTCTAAATACA

TTCAAATATGTATCCGCTCATGAGACAATAACCCTGATAAATGCTTCAATAATATTGAAAAAGGA

AGAGTATGAGTATTCAACATTTCCGTGTCGCCCTTATTCCCTTTTTTGCGGCATTTTGCCTTCCTGT

TTTTGCTCACCCAGAAACGCTGGTGAAAGTAAAAGATGCTGAAGATCAGTTGGGTGCACGAGTG

GGTTACATCGAACTGGATCTCAACAGCGGTAAGATCCTTGAGAGTTTTCGCCCCGAAGAACGTTT

TCCAATGATGAGCACTTTTAAAGTTCTGCTATGTGGCGCGGTATTATCCCGTATTGACGCCGGGCA

AGAGCAACTCGGTCGCCGCATACACTATTCTCAGAATGACTTGGTTGAGTACTCACCAGTCACA

GAAAAGCATCTTACGGATGGCATGACAGTAAGAGAATTATGCAGTGCTGCCATAACCATGAGTG

ATAACACTGCGGCCAACTTACTTCTGACAACGATCGGAGGACCGAAGGAGCTAACCGCTTTTTT

GCACAACATGGGGGATCATGTAACTCGCCTTGATCGTTGGGAACCGGAGCTGAATGAAGCCATA

CCAAACGACGAGCGTGACACCACGATGCCTGTAGCAATGGCAACAACGTTGCGCAAACTATTA

ACTGGCGAACTACTTACTCTAGCTTCCCGGCAACAATTAATAGACTGGATGGAGGCGGATAAAG

TTGCAGGACCACTTCTGCGCTCGGCCCTTCCGGCTGGCTGGTTTATTGCTGATAAATCTGGAGCC

GGTGAGCGTGGGTCTCGCGGTATCATTGCAGCACTGGGGCCAGATGGTAAGCCCTCCCGTATCGT

AGTTATCTACACGACGGGGAGTCAGGCAACTATGGATGAACGAAATAGACAGATCGCTGAGATA

GGTGCCTCACTGATTAAGCATTGGTAACTGTCAGACCAAGTTTACTCATATATACTTTAGATTGAT

TTAAAACTTCATTTTTAATTTAAAAGGATCTAGGTGAAGATCCTTTTTGATAATCTCATGACCAAA

ATCCCTTAACGTGAGTTTTCGTTCCACTGAGCGTCAGACCCCGTAGAAAAGATCAAAGGATCTTC

TTGAGATCCTTTTTTTCTGCGCGTAATCTGCTGCTTGCAAACAAAAAAACCACCGCTACCAGCGG

TGGTTTGTTTGCCGGATCAAGAGCTACCAACTCTTTTTCCGAAGGTAACTGGCTTCAGCAGAGCG

CAGATACCAAATACTGTTCTTCTAGTGTAGCCGTAGTTAGGCCACCACTTCAAGAACTCTGTAGC

ACCGCCTACATACCTCGCTCTGCTAATCCTGTTACCAGTGGCTGCTGCCAGTGGCGATAAGTCGT

GTCTTACCGGGTTGGACTCAAGACGATAGTTACCGGATAAGGCGCAGCGGTCGGGCTGAACGGG

GGGTTCGTGCACACAGCCCAGCTTGGAGCGAACGACCTACACCGAACTGAGATACCTACAGCG

TGAGCTATGAGAAAGCGCCACGCTTCCCGAAGGGAGAAAGGCGGACAGGTATCCGGTAAGCGG

CAGGGTCGGAACAGGAGAGCGCACGAGGGAGCTTCCAGGGGGAAACGCCTGGTATCTTTATAG

TCCTGTCGGGTTTCGCCACCTCTGACTTGAGCGTCGATTTTTGTGATGCTCGTCAGGGGGGCGGA

GCCTATGGAAAAACGCCAGCAACGCGGCCTTTTTACGGTTCCTGGCCTTTTGCTGGCCTTTTGCT

CACATGGCTCGAC

5. p62 WT-hRluc-Fluc

AGATCTGCGCAGCACCATGGCCTGAAATAACCTCTGAAAGAGGAACTTGGTTAGGTACCTTCTG

AGGCGGAAAGAACCAGCTGTGGAATGTGTGTCAGTTAGGGTGTGGAAAGTCCCCAGGCTCCCC

AGCAGGCAGAAGTATGCAAAGCATGCATCTCAATTAGTCAGCAACCAGGTGTGGAAAGTCCCC

AGGCTCCCCAGCAGGCAGAAGTATGCAAAGCATGCATCTCAATTAGTCAGCAACCATAGTCCC

GCCCCTAACTCCGCCCATCCCGCCCCTAACTCCGCCCAGTTCCGCCCATTCTCCGCCCCATGGCT

GACTAATTTTTTTTATTTATGCAGAGGCCGAGGCCGCCTCGGCCTCTGAGCTATTCCAGAAGTAG

TGAGGAGGCTTTTTTGGAGGCCTAGGCTTTTGCAAAAAGCTTGATTCTTCTGACACAACAGTCTC

GAACTTAAGCTGCAGAAGTTGGTCGTGAGGCACTGGGCAGGTAAGTATCAAGGTTACAAGACA

GGTTTAAGGAGACCAATAGAAACTGGGCTTGTCGAGACAGAGAAGACTCTTGCGTTTCTGATA

GGCACCTATTGGTCTTACTGACATCCACTTTGCCTTTCTCTCCACAGGTGTCCACTCCCAGTTCAA

TTACAGCTCTTAAGGCTAGAGTACTTAATACGACTCACTATAGGCTAGCCACCATGGCTTCCAA

GGTGTACGACCCCGAGCAACGCAAACGCATGATCACTGGGCCTCAGTGGTGGGCTCGCTGCAA

GCAAATGAACGTGCTGGACTCCTTCATCAACTACTATGATTCCGAGAAGCACGCCGAGAACGC

CGTGATTTTTCTGCATGGTAACGCTGCCTCCAGCTACCTGTGGAGGCACGTCGTGCCTCACATCG

AGCCCGTGGCTAGATGCATCATCCCTGATCTGATCGGAATGGGTAAGTCCGGCAAGAGCGGGA

ATGGCTCATATCGCCTCCTGGATCACTACAAGTACCTCACCGCTTGGTTCGAGCTGCTGAACCTT

CCAAAGAAAATCATCTTTGTGGGCCACGACTGGGGGGCTTGTCTGGCCTTTCACTACTCCTACG

AGCACCAAGACAAGATCAAGGCCATCGTCCATGCTGAGAGTGTCGTGGACGTGATCGAGTCCT

GGGACGAGTGGCCTGACATCGAGGAGGATATCGCCCTGATCAAGAGCGAAGAGGGCGAGAAA

ATGGTGCTTGAGAATAACTTCTTCGTCGAGACCATGCTCCCAAGCAAGATCATGCGGAAACTGG

AGCCTGAGGAGTTCGCTGCCTACCTGGAGCCATTCAAGGAGAAGGGCGAGGTTAGACGGCCTA

CCCTCTCCTGGCCTCGCGAGATCCCTCTCGTTAAGGGAGGCAAGCCCGACGTCGTCCAGATTGT

CCGCAACTACAACGCCTACCTTCGGGCCAGCGACGATCTGCCTAAGATGTTCATCGAGTCCGAC

CCTGGGTTCTTTTCCAACGCTATTGTCGAGGGAGCTAAGAAGTTCCCTAACACCGAGTTCGTGA

AGGTGAAGGGCCTCCACTTCAGCCAGGAGGACGCTCCAGATGAAATGGGTAAGTACATCAAGA

GCTTCGTGGAGCGCGTGCTGAAGAACGAGCAGTAAATGGCGTCGTTCACGGTGAAGGCCTATC

TTCTGGGCAAGGAGGAGGCGACCCGCGAGATCCGCCGCTTCAGCTTCTGCTTCAGCCCGGAGCC

GGAGGCGGAAGCCCAAGCCGCGGCCGGCCCGGGGCCCTGCGAGAGGCTGCTGAGCCGAGTGG

CTGTGCTGTTCCCCACGCTGAGGCCTGGCGGCTTCCAGGCGCACTACCGCGATGAGGATGGGGA

CTTGGTTGCCTTTTCCAGTGATGAGGAGCTGACAATGGCTATGTCCTATGTGAAAGATGACATCT

TCCGCATCTACATTAAAGAGAAGAAGGAGTGCCGGCGGGAACATCGCCCACCATGTGCTCAGG

AGGCACCCCGAAACATGGTGCACCCCAATGTGATCTGTGATGGTTGCAACGGGCCTGTGGTGG

GAACTCGCTATAAGTGCAGTGTGTGCCCAGACTACGACCTGTGCAGCGTGTGCGAGGGGAAGG

GCCTGCACAGGGAACACAGCAAGCTCATCTTTCCCAACCCCTTTGGCCACCTCTCTGATAGCTT

CTCTCATAGCCGCTGGCTTCGGAAGCTGAAACATGGACACTTTGGCTGGCCTGGCTGGGAGATG

GGCCCACCGGGGAACTGGAGCCCACGTCCTCCTCGTGCAGGGGATGGCCGCCCTTGCCCTACA

GCTGAGTCAGCTTCTGCTCCACCAGAAGATCCCAATGTCAATTTCCTGAAGAATGTGGGGGAGA

GTGTGGCAGCTGCCCTCAGCCCTCTAGGCATTGAGGTTGACATTGATGTGGAACATGGAGGGAA

GAGAAGCCGCCTGACACCCACTACCCCAGAAAGTTCCAGCACAGGCACAGAAGACAAGAGTA

ACACTCAGCCAAGCAGCTGCTCTTCGGAAGTCAGCAAACCTGACGGGGCTGGGGAGGGCCCTG

CTCAGTCTCTGACAGAGCAAATGAAAAAGATAGCCTTGGAGTCGGTGGGACAGCCAGAGGAA

CAGATGGAGTCGGGAAACTGCTCAGGAGGAGACGATGACTGGACACATTTGTCTTCAAAAGAA

GTGGACCCATCTACAGAGGCTGATCCCCGGCTGATTGAGTCCCTCTCCCAGATGCTGTCCATGG

GTTTCTCGGATGAAGGCGGCTGGCTCACCAGGCTCCTACAGACCAAGAATTACGACATCGGGG

CTGCTCTGGACACGATCCAGTATTCGAAGCACCCTCCACCATTGTGAAAGCTTGGCATTCCGGT

ACTGTTGGTAAAGCCACCATGGCCGATGCTAAGAACATTAAGAAGGGCCCTGCTCCCTTCTACC

CTCTGGAGGATGGCACCGCTGGCGAGCAGCTGCACAAGGCCATGAAGAGGTATGCCCTGGTGC

CTGGCACCATTGCCTTCACCGATGCCCACATTGAGGTGGACATCACCTATGCCGAGTACTTCGA

GATGTCTGTGCGCCTGGCCGAGGCCATGAAGAGGTACGGCCTGAACACCAACCACCGCATCGT

GGTGTGCTCTGAGAACTCTCTGCAGTTCTTCATGCCAGTGCTGGGCGCCCTGTTCATCGGAGTGG

CCGTGGCCCCTGCTAACGACATTTACAACGAGCGCGAGCTGCTGAACAGCATGGGCATTTCTCA

GCCTACCGTGGTGTTCGTGTCTAAGAAGGGCCTGCAGAAGATCCTGAACGTGCAGAAGAAGCT

GCCTATCATCCAGAAGATCATCATCATGGACTCTAAGACCGACTACCAGGGCTTCCAGAGCATG

TACACATTCGTGACATCTCATCTGCCTCCTGGCTTCAACGAGTACGACTTCGTGCCAGAGTCTTT

CGACAGGGACAAAACCATTGCCCTGATCATGAACAGCTCTGGGTCTACCGGCCTGCCTAAGGG

CGTGGCCCTGCCTCATCGCACCGCCTGTGTGCGCTTCTCTCACGCCCGCGACCCTATTTTCGGCA

ACCAGATCATCCCCGACACCGCTATTCTGAGCGTGGTGCCATTCCACCACGGCTTCGGCATGTT

CACCACCCTGGGCTACCTGATTTGCGGCTTTCGGGTGGTGCTGATGTACCGCTTCGAGGAGGAG

CTGTTCCTGCGCAGCCTGCAAGACTACAAAATTCAGTCTGCCCTGCTGGTGCCAACCCTGTTCA

GCTTCTTCGCTAAGAGCACCCTGATCGACAAGTACGACCTGTCTAACCTGCACGAGATTGCCTC

TGGCGGCGCCCCACTGTCTAAGGAGGTGGGCGAAGCCGTGGCCAAGCGCTTTCATCTGCCAGG

CATCCGCCAGGGCTACGGCCTGACCGAGACAACCAGCGCCATTCTGATTACCCCAGAGGGCGA

CGACAAGCCTGGCGCCGTGGGCAAGGTGGTGCCATTCTTCGAGGCCAAGGTGGTGGACCTGGA

CACCGGCAAGACCCTGGGAGTGAACCAGCGCGGCGAGCTGTGTGTGCGCGGCCCTATGATTAT

GTCCGGCTACGTGAATAACCCTGAGGCCACAAACGCCCTGATCGACAAGGACGGCTGGCTGCA

CTCTGGCGACATTGCCTACTGGGACGAGGACGAGCACTTCTTCATCGTGGACCGCCTGAAGTCT

CTGATCAAGTACAAGGGCTACCAGGTGGCCCCAGCCGAGCTGGAGTCTATCCTGCTGCAGCAC

CCTAACATTTTCGACGCCGGAGTGGCCGGCCTGCCCGACGACGATGCCGGCGAGCTGCCTGCC

GCCGTCGTCGTGCTGGAACACGGCAAGACCATGACCGAGAAGGAGATCGTGGACTATGTGGCC

AGCCAGGTGACAACCGCCAAGAAGCTGCGCGGCGGAGTGGTGTTCGTGGACGAGGTGCCCAA

GGGCCTGACCGGCAAGCTGGACGCCCGCAAGATCCGCGAGATCCTGATCAAGGCTAAGAAAG

GCGGCAAGATCGCCGTGTAATAATTCTAGAGTCGGGGCGGCCGGCCGCTTCGAGCAGACATGA

TAAGATACATTGATGAGTTTGGACAAACCACAACTAGAATGCAGTGAAAAAAATGCTTTATTTG

TGAAATTTGTGATGCTATTGCTTTATTTGTAACCATTATAAGCTGCAATAAACAAGTTAACAACA

ACAATTGCATTCATTTTATGTTTCAGGTTCAGGGGGAGGTGTGGGAGGTTTTTTAAAGCAAGTAA

AACCTCTACAAATGTGGTAAAATCGATAAGGATCCAGGTGGCACTTTTCGGGGAAATGTGCGC

GGAACCCCTATTTGTTTATTTTTCTAAATACATTCAAATATGTATCCGCTCATGAGACAATAACC

CTGATAAATGCTTCAATAATATTGAAAAAGGAAGAGTATGAGTATTCAACATTTCCGTGTCGCC

CTTATTCCCTTTTTTGCGGCATTTTGCCTTCCTGTTTTTGCTCACCCAGAAACGCTGGTGAAAGTA

AAAGATGCTGAAGATCAGTTGGGTGCACGAGTGGGTTACATCGAACTGGATCTCAACAGCGGT

AAGATCCTTGAGAGTTTTCGCCCCGAAGAACGTTTTCCAATGATGAGCACTTTTAAAGTTCTGCT

ATGTGGCGCGGTATTATCCCGTATTGACGCCGGGCAAGAGCAACTCGGTCGCCGCATACACTAT

TCTCAGAATGACTTGGTTGAGTACTCACCAGTCACAGAAAAGCATCTTACGGATGGCATGACAG

TAAGAGAATTATGCAGTGCTGCCATAACCATGAGTGATAACACTGCGGCCAACTTACTTCTGAC

AACGATCGGAGGACCGAAGGAGCTAACCGCTTTTTTGCACAACATGGGGGATCATGTAACTCG

CCTTGATCGTTGGGAACCGGAGCTGAATGAAGCCATACCAAACGACGAGCGTGACACCACGAT

GCCTGTAGCAATGGCAACAACGTTGCGCAAACTATTAACTGGCGAACTACTTACTCTAGCTTCC

CGGCAACAATTAATAGACTGGATGGAGGCGGATAAAGTTGCAGGACCACTTCTGCGCTCGGCC

CTTCCGGCTGGCTGGTTTATTGCTGATAAATCTGGAGCCGGTGAGCGTGGGTCTCGCGGTATCAT

TGCAGCACTGGGGCCAGATGGTAAGCCCTCCCGTATCGTAGTTATCTACACGACGGGGAGTCA

GGCAACTATGGATGAACGAAATAGACAGATCGCTGAGATAGGTGCCTCACTGATTAAGCATTG

GTAACTGTCAGACCAAGTTTACTCATATATACTTTAGATTGATTTAAAACTTCATTTTTAATTTAA

AAGGATCTAGGTGAAGATCCTTTTTGATAATCTCATGACCAAAATCCCTTAACGTGAGTTTTCGT

TCCACTGAGCGTCAGACCCCGTAGAAAAGATCAAAGGATCTTCTTGAGATCCTTTTTTTCTGCGC

GTAATCTGCTGCTTGCAAACAAAAAAACCACCGCTACCAGCGGTGGTTTGTTTGCCGGATCAAG

AGCTACCAACTCTTTTTCCGAAGGTAACTGGCTTCAGCAGAGCGCAGATACCAAATACTGTTCT

TCTAGTGTAGCCGTAGTTAGGCCACCACTTCAAGAACTCTGTAGCACCGCCTACATACCTCGCT

CTGCTAATCCTGTTACCAGTGGCTGCTGCCAGTGGCGATAAGTCGTGTCTTACCGGGTTGGACTC

AAGACGATAGTTACCGGATAAGGCGCAGCGGTCGGGCTGAACGGGGGGTTCGTGCACACAGCC

CAGCTTGGAGCGAACGACCTACACCGAACTGAGATACCTACAGCGTGAGCTATGAGAAAGCGC
CACGCTTCCCGAAGGGAGAAAGGCGGACAGGTATCCGGTAAGCGGCAGGGTCGGAACAGGAG
AGCGCACGAGGGAGCTTCCAGGGGGAAACGCCTGGTATCTTTATAGTCCTGTCGGGTTTCGCCA
CCTCTGACTTGAGCGTCGATTTTTGTGATGCTCGTCAGGGGGGCGGAGCCTATGGAAAAACGCC
AGCAACGCGGCCTTTTTACGGTTCCTGGCCTTTTGCTGGCCTTTTGCTCACATGGCTCGAC

6. p62 MUT-hRluc-Fluc

AGATCTGCGCAGCACCATGGCCTGAAATAACCTCTGAAAGAGGAACTTGGTTAGGTACCTTCTG
AGGCGGAAAGAACCAGCTGTGGAATGTGTGTCAGTTAGGGTGTGGAAAGTCCCCAGGCTCCCC
AGCAGGCAGAAGTATGCAAAGCATGCATCTCAATTAGTCAGCAACCAGGTGTGGAAAGTCCCC
AGGCTCCCCAGCAGGCAGAAGTATGCAAAGCATGCATCTCAATTAGTCAGCAACCATAGTCCC
GCCCCTAACTCCGCCCATCCCGCCCCTAACTCCGCCCAGTTCCGCCCATTCTCCGCCCCATGGCT
GACTAATTTTTTTTATTTATGCAGAGGCCGAGGCCGCCTCGGCCTCTGAGCTATTCCAGAAGTAG
TGAGGAGGCTTTTTTGGAGGCCTAGGCTTTTGCAAAAAGCTTGATTCTTCTGACACAACAGTCTC
GAACTTAAGCTGCAGAAGTTGGTCGTGAGGCACTGGGCAGGTAAGTATCAAGGTTACAAGACA
GGTTTAAGGAGACCAATAGAAACTGGGCTTGTCGAGACAGAGAAGACTCTTGCGTTTCTGATA
GGCACCTATTGGTCTTACTGACATCCACTTTGCCTTTCTCTCCACAGGTGTCCACTCCCAGTTCAA
TTACAGCTCTTAAGGCTAGAGTACTTAATACGACTCACTATAGGCTAGCCACCATGGCTTCCAA
GGTGTACGACCCCGAGCAACGCAAACGCATGATCACTGGGCCTCAGTGGTGGGCTCGCTGCAA
GCAAATGAACGTGCTGGACTCCTTCATCAACTACTATGATTCCGAGAAGCACGCCGAGAACGC
CGTGATTTTTCTGCATGGTAACGCTGCCTCCAGCTACCTGTGGAGGCACGTCGTGCCTCACATCG
AGCCCGTGGCTAGATGCATCATCCCTGATCTGATCGGAATGGGTAAGTCCGGCAAGAGCGGGA
ATGGCTCATATCGCCTCCTGGATCACTACAAGTACCTCACCGCTTGGTTCGAGCTGCTGAACCTT
CCAAAGAAAATCATCTTTGTGGGCCACGACTGGGGGGCTTGTCTGGCCTTTCACTACTCCTACG
AGCACCAAGACAAGATCAAGGCCATCGTCCATGCTGAGAGTGTCGTGGACGTGATCGAGTCCT
GGGACGAGTGGCCTGACATCGAGGAGGATATCGCCCTGATCAAGAGCGAAGAGGGCGAGAAA
ATGGTGCTTGAGAATAACTTCTTCGTCGAGACCATGCTCCCAAGCAAGATCATGCGGAAACTGG
AGCCTGAGGAGTTCGCTGCCTACCTGGAGCCATTCAAGGAGAAGGGCGAGGTTAGACGGCCTA
CCCTCTCCTGGCCTCGCGAGATCCCTCTCGTTAAGGGAGGCAAGCCCGACGTCGTCCAGATTGT
CCGCAACTACAACGCCTACCTTCGGGCCAGCGACGATCTGCCTAAGATGTTCATCGAGTCCGAC

CCTGGGTTCTTTTCCAACGCTATTGTCGAGGGAGCTAAGAAGTTCCCTAACACCGAGTTCGTGA

AGGTGAAGGGCCTCCACTTCAGCCAGGAGGACGCTCCAGATGAAATGGGTAAGTACATCAAGA

GCTTCGTGGAGCGCGTGCTGAAGAACGAGCAGTAAATGGCGTCGTTCACGGTGAAGGCCTATC

TTCTGGGCAAGGAGGAGGCGACCCGCGAGATCCGCCGCTTCAGCTTCTGCTTCAGCCCGGAGCC

GGAGGCGGAAGCCCAAGCCGCGGCCGGCCCGGGGCCCTGCGAGAGGCTGCTGAGCCGAGTGG

CTGTGCTGTTCCCCACGCTGAGGCCTGGCGGCTTCCAGGCGCACTACCGCGATGAGGATGGGGA

CTTGGTTGCCTTTTCCAGTGATGAGGAGCTGACAATGGCTATGTCCTATGTGAAAGATGACATCT

TCCGCATCTACATTAAAGAGAAGAAGGAGTGCCGGCGGGAACATCGCCCACCATGTGCTCAGG

AGGCACCCCGAAACATGGTGCACCCCAATGTGATCTGTGATGGTTGCAACGGGCCTGTGGTGG

GAACTCGCTATAAGTGCAGTGTGTGCCCAGACTACGACCTGTGCAGCGTGTGCGAGGGGAAGG

GCCTGCACAGGGAACACAGCAAGCTCATCTTTCCCAACCCCTTTGGCCACCTCTCTGATAGCTT

CTCTCATAGCCGCTGGCTTCGGAAGCTGAAACATGGACACTTTGGCTGGCCTGGCTGGGAGATG

GGCCCACCGGGGAACTGGAGCCCACGTCCTCCTCGTGCAGGGGATGGCCGCCCTTGCCCTACA

GCTGAGTCAGCTTCTGCTCCACCAGAAGATCCCAATGTCAATTTCCTGAAGAATGTGGGGGAGA

GTGTGGCAGCTGCCCTCAGCCCTCTAGGCATTGAGGTTGACATTGATGTGGAACATGGAGGGAA

GAGAAGCCGCCTGACACCCACTACCCCAGAAAGTTCCAGCACAGGCACAGAAGACAAGAGTA

ACACTCAGCCAAGCAGCTGCTCTTCGGAAGTCAGCAAACCTGACGGGGCTGGGGAGGCGGGAC

GTGTCTCAGACTGAGAGCAAATGAAAAAGATAGCCTTGGAGTCGGTGGGACAGCCAGAGGAA

CAGATGGAGTCGGGAAACTGCTCAGGAGGAGACGATGACTGGACACATTTGTCTTCAAAAGAA

GTGGACCCATCTACAGAGGCTGATCCCCGGCTGATTGAGTCCCTCTCCCAGATGCTGTCCATGG

GTTTCTCGGATGAAGGCGGCTGGCTCACCAGGCTCCTACAGACCAAGAATTACGACATCGGGG

CTGCTCTGGACACGATCCAGTATTCGAAGCACCCTCCACCATTGTGAAAGCTTGGCATTCCGGT

ACTGTTGGTAAAGCCACCATGGCCGATGCTAAGAACATTAAGAAGGGCCCTGCTCCCTTCTACC

CTCTGGAGGATGGCACCGCTGGCGAGCAGCTGCACAAGGCCATGAAGAGGTATGCCCTGGTGC

CTGGCACCATTGCCTTCACCGATGCCCACATTGAGGTGGACATCACCTATGCCGAGTACTTCGA

GATGTCTGTGCGCCTGGCCGAGGCCATGAAGAGGTACGGCCTGAACACCAACCACCGCATCGT

GGTGTGCTCTGAGAACTCTCTGCAGTTCTTCATGCCAGTGCTGGGCGCCCTGTTCATCGGAGTGG

CCGTGGCCCCTGCTAACGACATTTACAACGAGCGCGAGCTGCTGAACAGCATGGGCATTTCTCA

GCCTACCGTGGTGTTCGTGTCTAAGAAGGGCCTGCAGAAGATCCTGAACGTGCAGAAGAAGCT

GCCTATCATCCAGAAGATCATCATCATGGACTCTAAGACCGACTACCAGGGCTTCCAGAGCATG

TACACATTCGTGACATCTCATCTGCCTCCTGGCTTCAACGAGTACGACTTCGTGCCAGAGTCTTT

CGACAGGGACAAAACCATTGCCCTGATCATGAACAGCTCTGGGTCTACCGGCCTGCCTAAGGG

CGTGGCCCTGCCTCATCGCACCGCCTGTGTGCGCTTCTCTCACGCCCGCGACCCTATTTTCGGCA

ACCAGATCATCCCCGACACCGCTATTCTGAGCGTGGTGCCATTCCACCACGGCTTCGGCATGTT

CACCACCCTGGGCTACCTGATTTGCGGCTTTCGGGTGGTGCTGATGTACCGCTTCGAGGAGGAG

CTGTTCCTGCGCAGCCTGCAAGACTACAAAATTCAGTCTGCCCTGCTGGTGCCAACCCTGTTCA

GCTTCTTCGCTAAGAGCACCCTGATCGACAAGTACGACCTGTCTAACCTGCACGAGATTGCCTC

TGGCGGCGCCCCACTGTCTAAGGAGGTGGGCGAAGCCGTGGCCAAGCGCTTTCATCTGCCAGG

CATCCGCCAGGGCTACGGCCTGACCGAGACAACCAGCGCCATTCTGATTACCCCAGAGGGCGA

CGACAAGCCTGGCGCCGTGGGCAAGGTGGTGCCATTCTTCGAGGCCAAGGTGGTGGACCTGGA

CACCGGCAAGACCCTGGGAGTGAACCAGCGCGGCGAGCTGTGTGTGCGCGGCCCTATGATTAT

GTCCGGCTACGTGAATAACCCTGAGGCCACAAACGCCCTGATCGACAAGGACGGCTGGCTGCA

CTCTGGCGACATTGCCTACTGGGACGAGGACGAGCACTTCTTCATCGTGGACCGCCTGAAGTCT

CTGATCAAGTACAAGGGCTACCAGGTGGCCCCAGCCGAGCTGGAGTCTATCCTGCTGCAGCAC

CCTAACATTTTCGACGCCGGAGTGGCCGGCCTGCCCGACGACGATGCCGGCGAGCTGCCTGCC

GCCGTCGTCGTGCTGGAACACGGCAAGACCATGACCGAGAAGGAGATCGTGGACTATGTGGCC

AGCCAGGTGACAACCGCCAAGAAGCTGCGCGGCGGAGTGGTGTTCGTGGACGAGGTGCCCAA

GGGCCTGACCGGCAAGCTGGACGCCCGCAAGATCCGCGAGATCCTGATCAAGGCTAAGAAAG

GCGGCAAGATCGCCGTGTAATAATTCTAGAGTCGGGGCGGCCGGCCGCTTCGAGCAGACATGA

TAAGATACATTGATGAGTTTGGACAAACCACAACTAGAATGCAGTGAAAAAAATGCTTTATTTG

TGAAATTTGTGATGCTATTGCTTTATTTGTAACCATTATAAGCTGCAATAAACAAGTTAACAACA

ACAATTGCATTCATTTTATGTTTCAGGTTCAGGGGGAGGTGTGGGAGGTTTTTTAAAGCAAGTAA

AACCTCTACAAATGTGGTAAAATCGATAAGGATCCAGGTGGCACTTTTCGGGGAAATGTGCGC

GGAACCCCTATTTGTTTATTTTTCTAAATACATTCAAATATGTATCCGCTCATGAGACAATAACC

CTGATAAATGCTTCAATAATATTGAAAAAGGAAGAGTATGAGTATTCAACATTTCCGTGTCGCC

CTTATTCCCTTTTTTGCGGCATTTTGCCTTCCTGTTTTTGCTCACCCAGAAACGCTGGTGAAAGTA

AAAGATGCTGAAGATCAGTTGGGTGCACGAGTGGGTTACATCGAACTGGATCTCAACAGCGGT

AAGATCCTTGAGAGTTTTCGCCCCGAAGAACGTTTTCCAATGATGAGCACTTTTAAAGTTCTGCT

ATGTGGCGCGGTATTATCCCGTATTGACGCCGGGCAAGAGCAACTCGGTCGCCGCATACACTAT

TCTCAGAATGACTTGGTTGAGTACTCACCAGTCACAGAAAAGCATCTTACGGATGGCATGACAG

TAAGAGAATTATGCAGTGCTGCCATAACCATGAGTGATAACACTGCGGCCAACTTACTTCTGAC

AACGATCGGAGGACCGAAGGAGCTAACCGCTTTTTTGCACAACATGGGGGATCATGTAACTCG

CCTTGATCGTTGGGAACCGGAGCTGAATGAAGCCATACCAAACGACGAGCGTGACACCACGAT

GCCTGTAGCAATGGCAACAACGTTGCGCAAACTATTAACTGGCGAACTACTTACTCTAGCTTCC

CGGCAACAATTAATAGACTGGATGGAGGCGGATAAAGTTGCAGGACCACTTCTGCGCTCGGCC

CTTCCGGCTGGCTGGTTTATTGCTGATAAATCTGGAGCCGGTGAGCGTGGGTCTCGCGGTATCAT

TGCAGCACTGGGGCCAGATGGTAAGCCCTCCCGTATCGTAGTTATCTACACGACGGGGAGTCA

GGCAACTATGGATGAACGAAATAGACAGATCGCTGAGATAGGTGCCTCACTGATTAAGCATTG

GTAACTGTCAGACCAAGTTTACTCATATATACTTTAGATTGATTTAAAACTTCATTTTTAATTTAA

AAGGATCTAGGTGAAGATCCTTTTTGATAATCTCATGACCAAAATCCCTTAACGTGAGTTTTCGT

TCCACTGAGCGTCAGACCCCGTAGAAAAGATCAAAGGATCTTCTTGAGATCCTTTTTTTCTGCGC

GTAATCTGCTGCTTGCAAACAAAAAAACCACCGCTACCAGCGGTGGTTTGTTTGCCGGATCAAG

AGCTACCAACTCTTTTTCCGAAGGTAACTGGCTTCAGCAGAGCGCAGATACCAAATACTGTTCT

TCTAGTGTAGCCGTAGTTAGGCCACCACTTCAAGAACTCTGTAGCACCGCCTACATACCTCGCT

CTGCTAATCCTGTTACCAGTGGCTGCTGCCAGTGGCGATAAGTCGTGTCTTACCGGGTTGGACTC

AAGACGATAGTTACCGGATAAGGCGCAGCGGTCGGGCTGAACGGGGGGTTCGTGCACACAGCC

CAGCTTGGAGCGAACGACCTACACCGAACTGAGATACCTACAGCGTGAGCTATGAGAAAGCGC

CACGCTTCCCGAAGGGAGAAAGGCGGACAGGTATCCGGTAAGCGGCAGGGTCGGAACAGGAG

AGCGCACGAGGGAGCTTCCAGGGGGAAACGCCTGGTATCTTTATAGTCCTGTCGGGTTTCGCCA

CCTCTGACTTGAGCGTCGATTTTTGTGATGCTCGTCAGGGGGGCGGAGCCTATGGAAAAACGCC

AGCAACGCGGCCTTTTTACGGTTCCTGGCCTTTTGCTGGCCTTTTGCTCACATGGCTCGAC
